# Evoking Highly Immunogenic Ferroptosis Aided by Intramolecular Motion‐Induced Photo‐Hyperthermia for Cancer Therapy

**DOI:** 10.1002/advs.202104885

**Published:** 2022-02-08

**Authors:** Chao Chen, Zaiyu Wang, Shaorui Jia, Yuan Zhang, Shenglu Ji, Zheng Zhao, Ryan T. K. Kwok, Jacky W. Y. Lam, Dan Ding, Yang Shi, Ben Zhong Tang

**Affiliations:** ^1^ Department of Chemistry Hong Kong Branch of Chinese National Engineering Research Center for Tissue Restoration and Reconstruction State Key Laboratory of Molecular Nanoscience Division of Life Science Department of Chemical and Biological Engineering The Hong Kong University of Science and Technology Clear Water Bay Kowloon Hong Kong 999077 China; ^2^ Key Laboratory of Bioactive Materials Ministry of Education and College of Life Sciences Nankai University Tianjin 300071 China; ^3^ Department of Pharmaceutics School of Pharmacy Nanjing Medical University Nanjing 211116 China; ^4^ The Key Laboratory of Biomedical Material, School of Life Science and Technology Xinxiang Medical University Xinxiang 453003 China; ^5^ Shenzhen Institute of Molecular Aggregate Science and Technology School of Science and Engineering The Chinese University of Hong Kong Shenzhen 518172 China; ^6^ AIE Institute Guangzhou Development District, Huangpu Guangzhou 510530 China

**Keywords:** aggregate, ferroptosis, immunogenic cell death, intramolecular motion, photothermal therapy

## Abstract

Immunogenic cell death (ICD) through apoptosis or necroptosis is widely adopted to improve the therapeutic effect in cancer treatment by triggering a specific antitumor immunity. However, the tumor resistance to apoptosis/necroptosis seriously impedes the therapeutic effect. Recently, ferroptosis featured with excessive lipid peroxidation is demonstrated capable of bypassing the apoptosis/necroptosis resistance to kill cancer cells. To date, numerous efficient ferroptosis inducers are developed and successfully utilized for sensitizing cancer cells to ferroptosis. Unfortunately, these inducers can hardly generate adequate immunogenicity during induction of ferroptotic cancer cell death, which distinctly attenuates the efficacy of triggering antitumor immune response, therefore leads to unsatisfactory therapeutic effect. Herein, a novel high‐performance photothermal nanoparticle (TPA‐NDTA NP) is designed by exploiting energy via excited‐state intramolecular motion and employed for immensely assisting ferroptosis inducer to evoke highly efficient ICD through ferroptosis pathway. Tumor models with poor immunogenicity are used to demonstrate the tremendously enhanced therapeutic effect endowed by highly enhanced immunogenic ferroptosis in vitro and in vivo by virtue of the NPs. This study sheds new light on a previously unrecognized facet of boosting the immunogenicity of ferroptosis for achieving satisfactory therapeutic effect in cancer therapy.

## Graphical abstract

High‐performance photothermal nanoagents named TPA‐NDTA nanoparticles (NPs) are designed and fabricated by harvesting excited‐state intramolecular motion from strong‐donating molecular rotor using 1,2‐distearoyl‐*sn*‐glycero‐3‐phosphoethanolamine‐*N*‐[methoxy(polyethylene glycol)‐2000] as an encapsulation matrix. By virtue of the NP‐based photo‐hyperthermia, highly immunogenic ferroptosis is provoked, which leads to a specifically systemic antitumor immunity, therefore benefiting a lot to the ultimate therapeutic effect in ferroptosis‐driven cancer therapy.

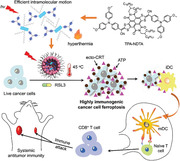

## 1 Introduction

The immunogenicity of cancer cell death has aroused wide attention in the field of cancer therapy as its critical role in triggering a specific antitumor immunity and improving the response rate during treatment combining with immune checkpoint inhibitors.^[^ ^1^ ^]^ Cancer cells undergoing immunogenic cell death (ICD) can elicit distinct damage‐associated molecular patterns (DAMPs) including surface‐exposed calreticulin (ecto‐CRT), high mobility group box 1 (HMGB1) and adenosine triphosphate (ATP).^[^ ^2^ ^]^ These released signals from dying cancer cells can unlock the intrinsic antitumor immune system by initiation of the immune recognition, promotion of the antigen presentation, and induction of a specific CD8^+^ T cell‐mediated immune response, benefitting a lot to cancer treatment.^[^ ^3^ ^]^ So far, efficient ICD is primarily induced through apoptosis or necroptosis.^[^ ^4^ ^]^ However, tumor resistance to these cell death pathways tremendously limits the therapeutic benefits via inducing ICD during cancer treatment.^[^ ^5^ ^]^ Thus, it is extremely desired to develop novel form of cancer cell death with high immunogenicity.

Ferroptosis is a newly defined iron‐dependent form of regulated cell death caused by excessive lipid peroxidation (LPO) and following membrane damage.^[^ ^6^ ^]^ According to recent studies, ferroptosis can be triggered by three ways: 1) inhibiting lipid repair system glutathione/glutathione peroxidase 4 (GSH/GPX4) complex to block the way of eliminating the harmful LPO, 2) inducing iron‐based Fenton reaction to increase the LPO by generating strongly oxidizing hydroxyl radical, and 3) increasing the level of ferroptosis‐relevant substrates by lipid metabolism.^[^ ^7^ ^]^ Taking the advantage of the exclusive cell death mechanism, ferroptosis has been demonstrated capable of bypassing the apoptosis/necroptosis resistance in cancer treatment.^[^ ^8^ ^]^ Moreover, ferroptosis has been found to be associated with inflammation response in some diseases, and can be used to fabricate cancer vaccine under certain conditions by releasing DAMPs, therefore brings great expectations to regain the immunological benefits of ICD for achieving satisfactory therapeutic effect in cancer treatment.^[^ ^9^ ^]^ Encouraged by this, various ferroptosis inducers have been identified and developed, such as inhibitors of GSH synthesis (erastin, sulfasalazine, sorafenib, etc.), GPX4 inhibitors (ras‐selective lethal small molecule 3 (RSL3), altretamine, etc.) and agents of increasing iron loading (ferrous chloride, hemin, salinomycin, various Fe‐based nanomaterials, etc.).^[^ ^10^ ^]^ Unfortunately, although these ferroptosis inducers have been verified capable of sensitizing cancer cells to ferroptotic cell death, there has been rarely proved that they are able to evoke highly immunogenic ferroptosis in cancer treatment, which vastly attenuates the immunological benefits, leading to compromised therapeutic effect during ferroptosis‐driven cancer therapy.^[^ ^8^, ^11^ ^]^ Therefore, it is of great significance to explore methods or agents to help the inducers eminently improve the immunogenicity of cancer cell death through ferroptosis pathway. However, there is scarcely related work to address this issue so far as we know.

Hyperthermia is the operation of heating the tumor‐loaded tissue to 41–46 °C,^[^ ^12^ ^]^ which has been successfully utilized as an “adjuvant” for improving the efficacy of chemotherapy, radiotherapy and ferroptosis therapy with little influence on surrounding normal tissues during treatment.^[^ ^13^ ^]^ More importantly, hyperthermia is closely related with the ICD induction, owing to the capability of causing endoplasmic reticulum (ER) stress,^[^ ^14^ ^]^ which partly results in the calreticulin exposure to the outer plasma membrane (ecto‐CRT, a key marker of ICD). Inspired by these findings, we wondered whether hyperthermia could be used to improve the immunogenicity of ferroptosis as well as enhance the efficacy for realizing impressive therapeutic effect in ferroptosis‐driven cancer treatment.

Among several strategies of generating hyperthermia (such as magnetic field hyperthermia, microwave hyperthermia, and ultrasound hyperthermia), photo‐hyperthermia (PHT) has received great attention due to the distinct advantages of economy, superb spatial‐temporal accuracy, and limited adverse effect.^[^ ^15^ ^]^ Thereby, many kinds of agents with efficient light‐to‐heat conversion have been developed to promote PHT performance, including inorganic materials (e.g., gold nanomaterials and metal chalcogenide), carbon materials, and organic materials such as indocyanine green (ICG), porphyrin and organic semiconducting polymers.^[^ ^16^ ^]^ Among them, organic materials attract more attention due to the good biosafety, adjustable structure, and facile functionalization, particularly in the form of nanoparticles, which exhibit distinct advantages in tumor targeting and accumulation.^[^ ^17^ ^]^ However, material design and preparation of high‐performance nanoagent with superb capability of photothermal conversion and excellent stability is rather limited.^[^ ^18^ ^]^ Very recently, we proposed a new strategy to design highly stable and efficient photothermal nanomaterials through harvesting energy via excited‐state intramolecular motion within nanoparticle.^[^ ^19^ ^]^ In this strategy, the intramolecular motion (from molecular rotor) enables the nanoparticle to utmost consumption of the absorbed energy through nonradiative heat dissipation, which is naturally suitable for building high photothermal conversion efficiency (PCE) nanoagents with excellent stability, therefore attracting great interest of researchers to regulate intramolecular motion to boost the effect of cancer photothermal therapy.^[^ ^20^ ^]^ Currently, although the excited‐state intramolecular motion of molecular rotor in nanoparticles for efficient heat generation has been demonstrated, the significance of electronic property of molecular rotor for achieving high PCE value and long absorption wavelength was yet systematically explored.

Herein, a novel photothermal agent named TPA‐NDTA was designed using a molecular rotor with strong electronic donor ability based on the strategy mentioned above. By virtue of the strong electronic donor property of molecular rotor in TPA‐NDTA, we obtained a powerful photothermal nanoagent (TPA‐NDTA NP) with high PCE (58.6%), strong and broad near infrared (NIR) absorption (from 700 to 1100 nm) and outstanding stability (including photostability, thermal and oxidative stability). The NPs were subsequently employed for generating PHT with a low laser power to highly improve the immunogenicity as well as the efficacy of RSL3‐induced ferroptosis, achieving an efficient systemic antitumor immunity and satisfactory therapeutic effect in mouse models bearing poor immunogenic 4T1 tumor. We declared for the first time that boosting the immunogenicity of ferroptotic cancer cell death is crucial for receiving great therapeutic benefits in ferroptosis‐based cancer therapy.

## 2 Results and Discussion

**Figure 1:**
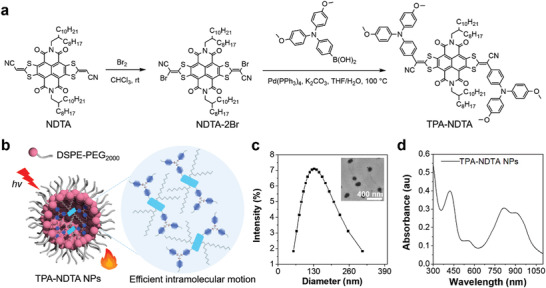
a) Synthetic route of compound TPA‐NDTA. b) Schematic of TPA‐NDTA NP using DSPE‐PEG_2000_ as an encapsulation matrix, displaying efficient excited‐state intramolecular motion of molecular rotor (the methoxy group substituted TPA) within NPs for heat generation under NIR laser irradiation. c) Dynamic light scattering (DLS) result shows the diameter of TPA‐NDTA NPs (20 µg mL^−1^ based on compound TPA‐NDTA). Inset is TEM image of the NPs. d) UV–vis absorption spectrum of TPA‐NDTA NPs solution in water (20 µg mL^−1^ based on compound TPA‐NDTA).

The compound TPA‐NDTA was designed to consist of three parts: 1) molecular rotor with strong electron‐donating ability (triphenylamine with methoxy group), 2) long alkyl chain that can provide enough space for efficient intramolecular motion, and 3) naphthalene diimide‐fused 2‐(1,3‐dithiol‐2‐ylidene) acetonitrile (NDTA) core,^[^ ^19^, ^21^ ^]^ which served as the electronic acceptor due to the strong electron‐withdrawing capability (**Figure** **1**a,b). To validate the molecular design concept, TPA‐NDTA was synthesized according to the synthetic route shown in Figure 1a and Figure S1 (Supporting Information). Briefly, NDTA was brominated by addition of bromine and chloroform in the room temperature to achieve compound NDTA‐2Br, which then reacted with boric acid of methoxy group substituted TPA to obtain compound TPA‐NDTA through Suzuki–Miyaura coupling reaction. The chemical structures of TPA‐NDTA and the intermediates were fully characterized by NMR and high‐resolution mass spectroscopy (Figures S2–S8, Supporting Information). Then, the theoretical calculation was performed by DFT methods at M06‐2X/Def2‐SVP level, Gaussian 16 program. As shown in Figure S9 (Supporting Information), the highest occupied molecular orbital (HOMO) mainly distributes on the methoxy group substituted TPA part, whereas the lowest unoccupied molecular orbital (LUMO) primarily localizes on NDTA part, which suggests the strong electron‐donating and withdrawing ability of the two parts, respectively. Moreover, the large dihedral angle between electronic donor (the methoxy group substituted TPA) and electronic acceptor (NDTA) is beneficial to the efficient intramolecular motion of the electronic donor moiety. Subsequently, we fabricated TPA‐NDTA NPs using an amphiphilic and biocompatible polymer 1,2‐distearoyl‐*sn*‐glycero‐3‐phosphoethanolamine‐*N*‐[methoxy(polyethylene glycol)‐2000] (DSPE‐PEG_2000_) as the matrix via simple nanoprecipitation method (Figure 1b). The average diameter, *Z*‐potential and morphology of TPA‐NDTA NPs were subsequently characterized by dynamic light scattering (DLS) and transmission electron microscopy (TEM), respectively. As displayed in Figure 1c, the NPs exhibit an average diameter of 132 nm and regular spherical shape with a slightly negative *Z*‐potential (−16.9 mV). The good colloid stability was also demonstrated by monitoring the diameter changes of the NPs in PBS solution within 7 days (Figure S10, Supporting Information). The strong and broad absorption of TPA‐NDTA NPs was observed in NIR range with a maximum wavelength of 820 nm (Figure 1d). Compared with the absorption profile in dilute THF solution (centered at 747 nm; Figure S11, Supporting Information), the maximum absorption of TPA‐NDTA NPs has an obvious red shift because of the increased molecular planarity within nanoparticles, which effectively covers the absorption range of the commercial 808 nm laser source. Furthermore, we found compound TPA‐NDTA is no emission in dilute THF solution and very dimly emissive within nanoparticles. These results suggest that even were confined in NPs, TPA‐NDTA molecules still undergo efficient excited‐state intramolecular motion to dissipate most absorbed energy through nonradiative heat generation (Figure S12, Supporting Information; Figure 1b), which guarantees the excellent photothermal conversion capability of the NPs. As the maximum absorption of TPA‐NDTA NPs is 820 nm and the minimum absorption of water also located in the range of 800–820 nm in the NIR region, commercial NIR laser of 808 nm therefore was selected to obtain the optimal photothermal performance of the NPs through maximally reducing the water influence in the following experiments.^[^ ^22^ ^]^

**Figure 2:**
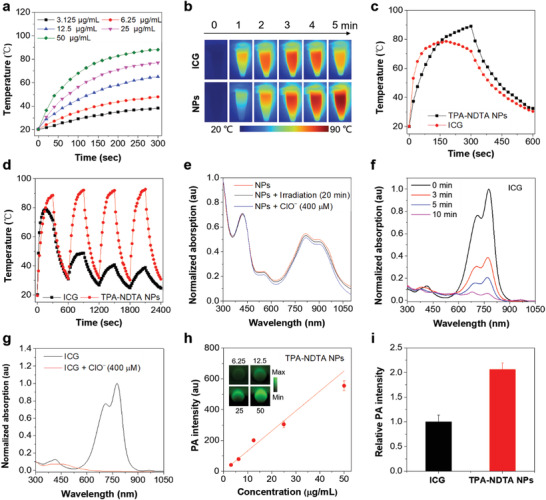
a) Photothermal performance of TPA‐NDTA NPs with a series of concentrations (3.125, 6.25, 12.5, 25, and 50 µg mL^−1^ based on compound TPA‐NDTA) under 808 nm laser irradiation (0.8 W cm^−2^). b) IR thermal images of ICG (50 µg mL^−1^) and TPA‐NDTA NPs solutions (50 µg mL^−1^ based on compound TPA‐NDTA) under different time points (0, 1, 2, 3, 4, and 5 min) of 808 nm laser irradiation (0.8 W cm^−2^). c) Temperature change profiles of ICG solution (50 µg mL^−1^) and TPA‐NDTA NPs solution (50 µg mL^−1^ based on compound TPA‐NDTA) under one heating–cooling process. For heating process, the samples were exposed to continuous 808 nm laser irradiation for 5 min (0.8 W cm^−2^), then the laser was turned off and the samples were cooling to environment temperature naturally for 5 min. d) Photostability and thermal stability of ICG solution (50 µg mL^−1^) and TPA‐NDTA NPs solution (50 µg mL^−1^ based on compound TPA‐NDTA) after four cycles of heating–cooling processes. e) Normalized absorption spectra of TPA‐NDTA NP solutions (12.5 µg mL^−1^ based on compound TPA‐NDTA) without any treatment (red), 20 min of 808 nm laser (0.8 W cm^−2^) irradiated NPs solution (dark), and ClO^−^ (400 × 10^−6^ m of the final concentration) added NPs solution (blue). f) Normalized absorption spectra of ICG solutions (12.5 µg mL^−1^) after 0, 3, 5, and 10 min of 808 nm laser irradiation (0.8 W cm^−2^). g) Normalized absorption spectra of ICG solutions (12.5 µg mL^−1^) with/without addition of ClO^− ^(400 × 10^−6^ m of the final concentration). h) PA intensities of TPA‐NDTA NPs solutions with different concentrations using MOST imaging system (inVision 256‐TF; iThera Medical, Germany) at 810 nm excitation. Inset: PA images of TPA‐NDTA NP solutions of different concentrations (6.25, 12.5, 25, and 50 µg mL^−1^ based on compound TPA‐NDTA) under 810 nm excitation using MOST imaging system. i) Quantitative analysis of relative PA intensity of ICG (100 × 10^−6^ m) and TPA‐NDTA NPs solution (100 × 10^−6^ m based on compound TPA‐NDTA) measured at 810 nm excitation using MOST imaging system.

We subsequently evaluated the photothermal conversion capability of TPA‐NDTA NPs with a series of concentrations under continuous 808 nm laser irradiation for 5 min. As shown in **Figure** **2**a, the temperatures quickly increased and then gradually reached the temperature plateau along with the irradiation time. Higher concentration of TPA‐NDTA NPs exhibited faster temperature increase. Subsequently, the clinical used efficient photothermal agent (ICG) and a NIR‐absorbing semiconducting polymer (PCPDTBT) were applied for comparison. As displayed in Figure 2b,c and Figure S13 (Supporting Information), TPA‐NDTA NPs reached the highest temperature of ≈90 °C upon sustaining 808 nm laser irradiation in 5 min, while the maximum temperatures of ICG and PCPDTBT NP solutions are ≈79 and ≈64 °C, respectively, under the same experimental conditions and mass concentration (50 µg mL^−1^). Moreover, after reaching the plateau, the temperature of ICG solution was gradually decreased, demonstrating that ICG cannot perform a stable heat output due to the poor thermal stability. In stark contrast, TPA‐NDTA NPs exhibit a superb performance of durably and steadily generate adequate photothermy, revealing the superiority of design strategy by harvesting energy via intramolecular motion from molecular rotor with strong electron‐donating ability. Then, the photothermal conversion efficiency (PCE) of TPA‐NDTA NPs (PCE = 58.6%) was measured and calculated according to the previous method.^[^ ^19^, ^23^ ^]^ Interestingly, compared with our previously reported photothermal molecule 2TPE‐NDTA that used TPE (tetraphenyl ethylene, relatively weak electron‐donor ability) as the molecular rotor (PCE = 43.0% in the form of nanoparticle), TPA‐NDTA NPs showed much higher value of PCE. Moreover, by virtue of the NIR absorption and high PCE, the temperature of the NP solution can be raised by ≈15 °C even covering a 5 mm thick chicken tissue under 808 nm laser irradiation (Figure S14, Supporting Information). Additionally, the maximum absorption of TPA‐NDTA NPs also shows over 100 nm redshift than that of 2TPE‐NDTA NPs. These comparisons clearly demonstrated the significance of electronic property of molecular rotor for PCE improvement and absorption extension when utilizing the energy from intramolecular motion to build photothermal NPs.

To further assess the photostability, thermal stability and oxidative stability, relevant experiments were conducted. As shown in Figure 2d, both TPA‐NDTA NPs and ICG were suffered to four cycles of laser on/off process (5 min of laser irradiation and 5 min of cooling down served as one cycle) to evaluate their photostability and thermal stability. From the result, TPA‐NDTA NPs solution can still be heated to ≈92 °C after three cycles, whereas ICG solution only could be heated to ≈40 °C much lower than that of fresh ICG solution (maximum temperature can reach ≈79 °C), revealing excellent photostability and thermal stability of TPA‐NDTA NPs. Furthermore, the absorption spectra of different TPA‐NDTA NPs solutions were measured before and after continuous 808 nm laser irradiation for 20 min. From the result in Figure 2e, there is no obvious change after long‐term laser irradiation. However, the significantly decreased absorption of ICG was occurred along with the irradiation time, owing to the severe degradation (Figure 2f). These results further verified the outstanding photostability and thermal stability of TPA‐NDTA NPs, demonstrating the success of building high‐performance nanoagents by the design strategy. It is known that tumor microenvironment is always featured with high level of reactive oxygen and nitrogen species (RONS), such as peroxynitrite (ONOO^−^), hydrogen peroxide (H_2_O_2_), hypochlorite (ClO^−^), etc. These excess RONS not only accelerate tumor progression, but also destroy the molecules delivered into tumor site.^[^ ^24^ ^]^ As depicted in Figure 2g, the absorption of ICG almost totally disappeared within 1 min after addition of ClO^−^ (at a final concentration of 400 × 10^−6^ m), suggesting that ICG was destroyed completely in the presence of ClO^−^. In contrast, the absorption of TPA‐NDTA NPs did not show obvious change with or without addition of ClO^−^, manifesting its outstanding oxidative stability (Figure 2e). Furthermore, Other kinds of RONS such as ONOO^− ^and H_2_O_2_ were also performed to evaluate the oxidative stability. As shown in Figure S15 (Supporting Information), compared with ICG solutions treated with ONOO^−^ or H_2_O_2_, the absorption spectra of the NPs solutions nearly no change after addition of these RONS, indicating the great oxidative stability. Encouraged by the superb photothermal conversion capability and ultrahigh stability of the NPs coupling with the great potential of photoacoustic (PA) imaging technique for clinical transformation,^[^ ^25^ ^]^ we next investigated the PA properties of the NPs. Figure S16 (Supporting Information) depicts the normalized PA spectrum of TPA‐NDTA NPs solution in the range of 680–900 nm, which corresponds well with its absorption. To further study the relationship between PA intensity and concentration of NPs, a series of TPA‐NDTA NPs solutions covering a concentration range from 3.125 to 50 µg mL^−1^ were prepared. As displayed in Figure 2h, there is a good liner relationship of PA signal intensity versus different concentrations of the NPs upon excitation at 810 nm. Moreover, compared with the efficient and widely used PA agent ICG, the NPs exhibited a 2.1 times higher PA intensity than that of ICG in the same molar concentration (Figure 2i). These results demonstrated the admirable capability of TPA‐NDTA NPs for PA imaging, which can be ascribed to the broad and high absorption in NIR region, outstanding photothermal conversion capability and ultrahigh stability.

After the detailed assessment of TPA‐NDTA NPs in terms of photothermal conversion capability, stability, and PA imaging property, we employed the NPs for generating adjuvant PHT to study whether it can significantly enhance the immunogenicity as well as the efficacy of ferroptosis in 4T1 murine breast cancer cells with poor immunogenicity. First, the cell viability of 4T1 cancer cells, 3T3 mouse fibroblast cells and LO2 human hepatocyte cells incubated with different concentrations of TPA‐NDTA NPs for 24 h or 72 h without laser irradiation were determined using the MTT (3‐(4,5‐dimethylthiazol‐2‐yl)‐2,5‐diphenyltetrazolium bromide) assay, respectively. As shown in Figures S17 and S18 (Supporting Information), no significant toxicity was observed in comparison with control group (without any treatment) even in the presence of 100 µg mL^−1^ of TPA‐NDTA NPs, confirming a good biocompatibility of the NPs in cellular level. Then, we fixed the concentration of TPA‐NDTA NPs at 25 µg mL^−1^ for 12 h incubation, irradiated the cells with 808 nm laser under different intensities for 5 min, and measured the cell viability after 24 h. From the MTT result in Figure S19 (Supporting Information), increased toxicity was observed along with the elevated laser intensity, 5 min of 808 nm laser irradiation at 0.45 W cm^−2^ (the cell viability was ≈90%) was selected to generate the PHT during following relevant experiments. Meanwhile, the killing efficacy of RSL3 in 4T1 cells was examined. RSL3 is one of the most widely used ferroptosis inducers due to its specific and direct inhibition of GPX4, which plays a central role in regulating ferroptosis by converting toxic LPO into nontoxic lipid alcohols. From the result of MTT assay in Figure S20 (Supporting Information), RSL3 can effectively kill 4T1 cells through ferroptosis pathway in a concentration‐dependent manner. Then, 5 × 10^−6^ m of RSL3 was chosen to explore the further enlargement of therapeutic effect via inducing highly immunogenic and efficient ferroptosis aid by PHT. Moreover, a specific ferroptosis inhibitor DFO (DFO can effectively inhibit the RSL3‐triggered ferroptosis) was introduced into the experiments to study whether the improved immunogenicity of ferroptosis as well as the efficacy were regulated by ferroptosis pathway.

**Figure 3:**
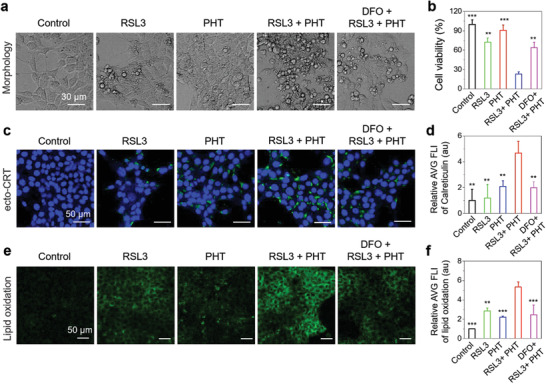
a) Representative images of cellular morphology of 4T1 cancer cells after 24 h of different treatments in five groups including “control,” “RSL3,” “PHT,” “RSL3 + PHT,” and “DFO + RSL3 + PHT” groups. b) Cell viability of 4T1 cancer cells in five groups exposed to corresponding treatments according to the design and detected using MTT assay after 24 h. c) Representative CLSM images and d) the quantitative analysis of ecto‐CRT of 4T1 cancer cells in five groups after 12 h of relevant treatments. Surface‐exposed calreticulin (ecto‐CRT) was detected by immunofluorescence staining with anticalreticulin primary antibody and Alexa Fluor 488‐conjugated secondary antibody using CLSM with 488 nm of excitation and 500–600 nm collection. e) Representative CLSM images and f) quantitative data of lipid peroxidation (LPO) in five groups after 12 h of different treatments. 10 × 10^−6^ m of C11‐BODIPY (a lipid peroxidation sensor) was added into the cells for 30 min, then the signals of the sensor were collected within 505–550 nm under 488 nm of excitation using CLSM. The green signals collected in this condition indicate the levels of LPO. MTT and quantitative data are presented as means ± SD and the statistical analysis method was one‐way ANOVA. *n* = 3 for each group. **P < 0.01, ***P < 0.001, in comparison with “RSL3 + PHT” group. NPs: 25 µg mL^−1^ based on compound TPA‐NDTA. Laser irradiation: 5 min, 0.45 W cm^−2^. RSL3: 5 × 10^−6^ m. DFO: 100 × 10^−6^ m.

Thus, 4T1 cancer cells were divided into five groups, namely “control,” “RSL3,” “PHT,” “RSL3 + PHT,” and “DFO + RSL3 + PHT,” respectively. For “RSL3 + PHT” group, the cells were incubated with TPA‐NDTA NPs (25 µg mL^−1^) for 12 h after cell adherence, then added 5 × 10^−6^ m of RSL3 into the culture medium. After 4 h, the cells were administrated with 808 nm laser irradiation (0.45 W cm^−2^) for 5 min and incubated for another 24 h. For comparison, “RSL3” group was only treated with 5 × 10^−6^ m of RSL3, “PHT” group was not treated with RSL3, and “DFO + RSL3 + PHT” group was extra added 100 × 10^−6^ m of DFO before 1 h of RSL3 addition. The 4T1 cells without any treatment were used as “control” group and all groups were measured by MTT assay post 24 h of 808 nm laser irradiation in “RSL3 + PHT” group. As shown in **Figure** **3**b, the cell viability of “RSL3 + PHT” group was sharply decreased to 22.9% in comparison with that of “RSL3” group (72.2%) or “PHT” group (90.6%), individually, demonstrating highly efficient RSL3‐induced ferroptosis could be achieved by the PHT effect of TPA‐NDTA NPs. However, after addition of DFO, the killing efficacy in “DFO + RSL3 + PHT” group was significantly attenuated (cell viability: 64.1%), which indicated that the enhanced killing efficacy was closely mediated by ferroptosis pathway. Furthermore, the morphology of the 4T1 cells after the same treatments shown in Figure 3a also verifies the same conclusion.

After affirming the enhancement of ferroptosis efficacy, we next detailly studied the immunogenicity of ferroptosis aided by PHT via analyzing the markers of ICD such as ecto‐CRT and released ATP using 4T1 cancer cells. As shown in Figure 3c,d and Figure S21 (Supporting Information), compared with “RSL3” group and other groups, the expression level of ecto‐CRT and the amount of released ATP were both substantially enhanced in “RSL3 + PHT” group, suggesting much higher immunogenicity were obtained during ferroptotic cell death in assistant of PHT. In contrast, both levels of ecto‐CRT and released ATP were prominently declined when treatment with DFO, demonstrating that the highly enhanced immunogenicity of ferroptotic cell death aided by PHT is regulated by the signaling pathway of ferroptosis. Because LPO is the key feature and driving force of ferroptosis, the cellular level of LPO in five groups were next evaluated using commercial LPO sensor (C11 BODIPY) after different treatments. As shown in Figure 3e,f, the fluorescence signal in “RSL3 + PHT” group was significantly stronger than that of other groups, revealing an apparently elevated level of cellular LPO in “RSL3 + PHT” group. These results suggest that enhanced ferroptosis pathway facilitates the improvement of efficacy and immunogenicity of ferroptosis through combined treatment (RSL3 + PHT) in 4T1 cells. Moreover, according to literature reports, ROS are engaged in the process of generating LPO and capable of triggering ICD by causing ER stress. Therefore, we further analyzed the level of intracellular ROS using commercial ROS indicator (2′,7′‐dichlorodihydrofluorescein diacetate, DCF‐DA), which can be oxidized to green‐emissive DCF by ROS in living cells. As depicted in Figure S22 (Supporting Information), the highest intracellular level of ROS was observed in “RSL3 + PHT” group, manifesting increased cellular ROS may be an upstream driver of evoking highly immunogenic and efficient cancer cell death through ferroptosis pathway in this experiment.

Encouraged by these results in 4T1 cells, in vivo relevant experiments were conducted in 4T1 tumor‐bearing mice. All animal experiments in this work were performed under the guidelines set by Tianjin Committee of Use and Care of Laboratory Animals, and the overall project protocols were approved by the Animal Ethics Committee of Nankai University. Because the poor immunogenicity of 4T1 cells coupling with limited infiltrating CD8^+^ T cells, 4T1 murine breast tumor is considered as an immunogenic “cold” tumor with highly aggressive and immunological unresponsiveness.^[^ ^26^ ^]^ Thereby, mouse model bearing 4T1 tumor was selected to assess the therapeutic effect in vivo through evoking highly immunogenic ferroptosis. At the beginning, healthy BALB/c mice were intravenously administrated with 100 µL of TPA‐NDTA NPs solution (200 µg mL^−1^ in 1 × PBS, 4 times higher than that used in following tumor treatment) to examine the potential toxicity. After 7 days, the blood biochemistry assay, liver function test and histopathological analysis of major organs (kidney, liver and spleen) were performed on the NP‐injected mice, respectively. As shown in Figures S23 and S24 (Supporting Information), there is no significant impact on the healthy mice after treatment with the NPs, certifying its good biocompatibility in vivo. Then, the capabilities of PA imaging and photothermal generation were investigated in 4T1 tumor‐bearing mice after intravenous injection of TPA‐NDTA NPs (100 µL and 50 µg mL^−1^). As shown in Figure S25 (Supporting Information), after NP injection, the PA signals gradually increased within tumor region and reached a peak at 6 h, exhibiting an excellent ability of PA tumor imaging, which also reveals the optimal time point of intervention according to maximum accumulation of the NPs. Based on this result, heat generation was monitored by IR thermography at 6 h post TPA‐NDTA NP injection (100 µL and 50 µg mL^−1^) under continuous 808 nm laser irradiation (0.6 W cm^−2^) on the tumor region. From the result in Figure S26 (Supporting Information), the tumor temperature quickly increased to 60 °C in 10 min of NP‐injected group under continuous 808 nm laser irradiation (0.6 W cm^−2^) while the tumor temperature of saline‐injected group was only slightly elevated to 39 °C (below the lower limit of hyperthermia), which demonstrated the superb photothermal conversion capability and outstanding stability of TPA‐NDTA NPs after intravenous administration, displaying a great potential for cancer treatment in vivo.

**Figure 4:**
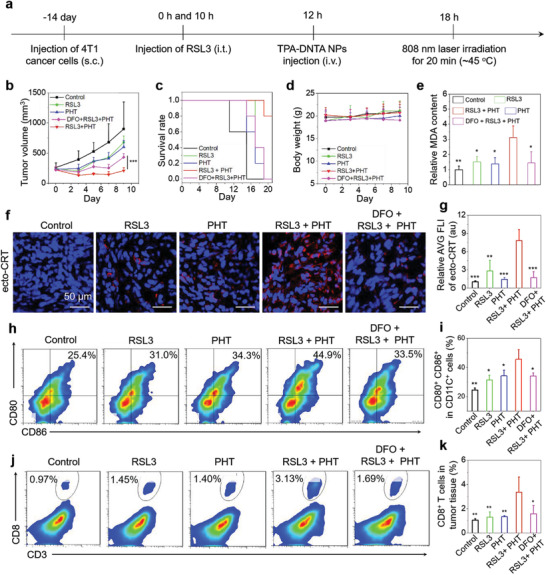
a) The procedure of building tumor model and combined treatment in the tumor‐bearing mice. (subcutaneous: s.c.; intratumor: i.t.; intravenous: i.v.). RSL3: 200 × 10^−6^ m. NPs: 50 µg mL^−1^ based on compound TPA‐NDTA, 100 µL. Laser irradiation: 808 nm, 20 min, 0.3 W cm^−2^. b) Tumor volume, c) survival rate, and d) body weight of the mice in five groups (“Control,” “RSL3,” “PHT,” “RSL3 + PHT,” and “DFO + RSL3 + PHT”) after different treatments. The tumor volume and body weight were recorded at the time points of 0, 3, 5, 7, and 9 days. The survival rate was monitored for 3 weeks. e) Relative MDA content of tumor tissues in five groups after 12 h of corresponding treatments. Malondialdehyde (MDA) is a natural product of lipid oxidation and the amount of MDA reveals the levels of LPO in vivo. f) Representative CLSM images and g) quantitative data of ecto‐CRT of tumor tissues in the five groups after 12 h of relevant treatments. The signals were detected by CLSM with excitation at 633 nm and collection from 650 to 750 nm. h) Representative flow cytometry data and i) quantitative data of DC maturation in lymph nodes after 2 days of different treatments. DC maturation is characterized by upregulated expression of CD80 and CD86. j) Representative flow cytometry data and k) quantitative data of CD8^+^ T cells (CD3^+^ and CD8^+^) in tumor tissues of five groups after 5 days of corresponding treatments. Data are presented as means ± SD. *n* = 3 for each group. The statistical analysis method was Student's *t*‐test in b) and one‐way ANOVA in (e), (g), (i), and (k). *P < 0.05, **P < 0.01, and ***P < 0.001, in comparison with “RSL3 + PHT” group.

After that, we estimated the therapeutic effect in vivo through evoking highly immunogenic and efficient ferroptosis aided by PHT using a mouse model bearing 4T1 tumor. Briefly, the tumor‐bearing mice were randomly divided into five groups, named the same as the cellular experiments (“Control,” “RSL3,” “PHT,” “RSL3 + PHT,” and “DFO + RSL3 +PHT”). The treatment procedure was depicted in **Figure** **4**a. After two weeks of tumor inoculation (1 × 10^5^ of live 4T1 cells), the mice were first received twice intratumor injection of RSL3 (25 µL, 200 × 10^−6^ m) at 0 and 10 h, then administrated with once intravenous injection of TPA‐NDTA NPs (100 µL and 50 µg mL^−1^) at 12 h, followed by treatment with 808 nm laser irradiation (0.3 W cm^−2^, at 18 h) to keep the tumor temperature at ≈45 °C for 20 min, subsequently recorded the tumor growth by caliper. It is noted that the effective PHT can be achieved by 808 nm laser irradiation with a relatively low power owing to the ultrahigh PCE and superb stability of the NPs, further reducing the side effect on surrounding tissues. As shown in Figure 4b, after the combined treatment, the tumor growth was obviously delayed in “RSL3 + PHT” group compared with that of “RSL3” group (without NP injection and 808 nm laser irradiation) and “PHT” group (without RLS3 administration), confirming a significantly improved therapeutic effect in ferroptosis‐driven cancer treatment through this method. In contrast, after treatment with DFO (50 µL, 400 × 10^−6^ m, once intratumor injection before 2 h of the first RSL3 administration), the inhibitory effect of tumor growth was distinctly weakened in “DFO + RSL3 + PHT” group, suggesting the enhanced antitumor effect is mediated through ferroptosis pathway. From the result of the survival rate in Figure 4c, 80% of treated mice in “RSL3 + PHT” group survived up to 21 days, much better than the therapeutic responses achieved in other groups, demonstrating the vastly improved therapeutic effect. The whole treatment process also exhibits negligible toxicity due to no obvious impact on the body weights of the mice during the treatment period (Figure 4d).

We subsequently analyzed the levels of ecto‐CRT and LPO in tumor tissues after 12 h of relevant treatments. As shown in Figure 4f,g, the expression levels of the key marker of ICD (ecto‐CRT) in “RSL3 + PHT” group is 2.9 times higher than that of “RSL3” group, and 4.6 times higher than that of “DFO + RSL3 + PHT” group, suggesting highly immunogenic ferroptosis was evoked by virtue of PHT and regulated by signaling pathway of ferroptosis. Then, the tumor LPO in five groups were measured using a commercial kit (Lipid Peroxidation MDA Assay Kit). As depicted in Figure 4e, the MDA content (revealed the LPO level) of “RSL3 + PHT” group is 2.1 and 2.2 times more than that of “RSL3” and “DFO + RSL3 + PHT” groups, respectively, indicating highly enhanced immunogenicity and efficacy of ferroptosis accompanied by the increasing LPO levels, which further drive the cancer cell death through ferroptosis pathway. It needs to be noted that only PHT treatment can neither trigger ferroptosis nor induce ICD in the experiments. These analyses demonstrated that employing PHT for evoking highly immunogenic and efficient ferroptosis plays a crucial role to obtain satisfactory therapeutic effect in ferroptosis‐based cancer treatment.

Different from nonimmunogenic cell death (non‐ICD), highly efficient ICD can substantially improve the therapeutic benefits of cancer treatment by provoking an effective antitumor immunity. During this immune response, released DAMPs such as ecto‐CRT from dying cancer cells stimulate the efficient antigen loading of dendritic cells (DCs) in tumor region, then the DCs migrate into the secondary lymphoid organ (e.g., lymph nodes) and become matured DCs by upregulating the costimulatory molecules (CD80^+^ and CD86^+^), finally the matured DCs present antigen information to T cells and prime a subsequently specific CD8^+^ T cell response.^[^ ^27^ ^]^ Similarly, highly immunogenic ferroptosis achieved by this method also should be able to effectively trigger this complete immune response. Accordingly, we analyzed the maturation of DCs in tumor draining lymph nodes after 2 days of corresponding treatments in five groups by flow cytometry. As displayed in Figure 4h,i, the proportion of matured DCs reached to 45.5% in “RSL3 + PHT” group, while the percentages were 24.4%, 31.3%, 34.2%, and 34.1% in “Control,” “RSL3,” “PHT,” and “DFO + RSL3 + PHT” groups, respectively, suggesting a more efficient tumor antigen presentation was obtained via evoking highly immunogenic ferroptosis aided by PHT. Next, infiltrating CD8^+^ T cells of tumor tissues in five groups were studied through flow cytometry analysis after 5 days of different treatments. As illustrated in Figure 4j,k, the proportion of CD8^+^ T cells in “RSL3 + PHT” group is 3.4, 2.6, 2.5, and 2.2 times higher than that of “Control,” “RSL3,” “PHT,” and “DFO + RSL3 + PHT” groups, demonstrating an efficient CD8^+^ T cell‐mediated antitumor immunity was provoked, which plays a critical role in delaying the tumor growth and changing the poor immunogenicity of “cold” 4T1 tumor, benefiting a lot to the finally admirable therapeutic effect.

**Figure 5:**
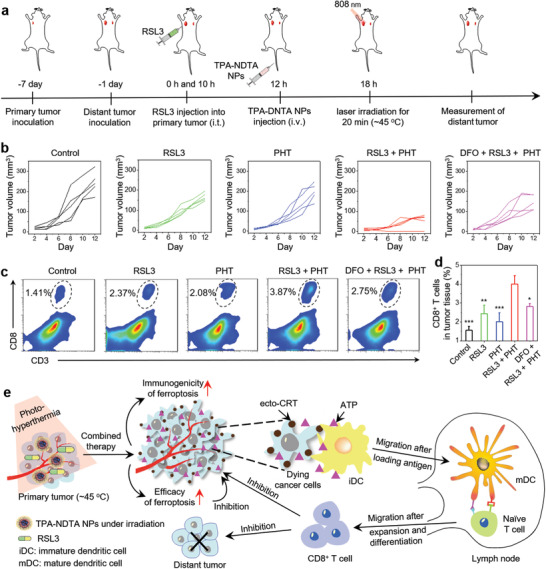
a) Schematic illustration of the procedure of building a bilateral tumor mouse model and combined treatment. Intratumor: i.t.; intravenous: i.v. RSL3 (injected into the primary tumor): 200 × 10^−6^ m. NPs: 50 µg mL^−1^ based on compound TPA‐NDTA, 100 µL. Laser irradiation (focused on primary tumor): 808 nm, 20 min, and 0.3 W cm^−2^. b) Tumor volume of the distant tumors in five groups (“Control,” “RSL3,” “PHT,” “RSL3 + PHT,” and “DFO + RSL3 + PHT”) after different treatments according to the design. c) Representative flow cytometry data and d) quantitative data of CD8^+^ T cells (CD3^+^ and CD8^+^) in distant tumor tissues of five groups after 10 days of corresponding treatments. Data are presented as means ± SD and the statistical analysis method was one‐way ANOVA. *n* = 3 for each group. *P < 0.05, **P < 0.01, and ***P < 0.001, in comparison with “RSL3 + PHT” group. e) Schematic illustration of antitumor mechanism of the combined treatment by highly boosting the immunogenicity and efficacy of RSL3‐induced ferroptosis under the assistance of TPA‐NDTA NP‐based PHT in a bilateral tumor mouse model. Released ATP and ecto‐CRT from dying cancer cells are the key markers of ICD. Red arrow means a high enhancement. iDC: immature dendritic cell. mDC: mature dendritic cell.

It is known that successful antitumor immunity usually exhibits a systemic protection effect against untreated tumors, which plays a pivotal role in attacking metastatic tumors, preventing tumor recurrence, and maintaining a long‐term antitumor effect.^[^ ^28^ ^]^ Therefore, we established a mouse model with bilateral 4T1 tumors in the axilla of mice to evaluate whether highly immunogenic ferroptosis can effectively stimulate a specifically systemic antitumor effect.^[^ ^29^ ^]^ The administration procedure was described in **Figure** **5**a. The heathy BALB/c mice were first inoculated with live 4T1 cancer cells (1 × 10^5^) into the right axilla of mice on day −7 to build the primary tumor, then established a distant tumor into the left axilla on day −1 by injection with the same 4T1 cancer cells (1 × 10^5^). Subsequently, the mice were randomly divided into five groups named as before. Then, the primary tumors of the mice were twice treated with RSL3 (200 × 10^−6^ m, 25 µL) by intratumor injection at 0 and 10 h, intravenously injected with TPA‐NDTA NPs (100 µL and 50 µg mL^−1^) at 12 h, followed by local treatment of 808 nm laser irradiation (0.3 W cm^−2^, at 18 h) on primary tumor to keep the tumor temperature at ≈45 °C for 20 min, finally monitored the distant tumor growth (without any treatment). As shown in Figure 5b, after the combined treatment, the distant tumor growth was apparently delayed in “RSL3 + PHT” group during the experimental period (the distant tumor disappeared in one mouse of this group), showing an impressive systemic antitumor effect compared with that of “Control” group (without treatment), “RSL3” group (without NP injection and laser irradiation), and “PHT” group (without RSL3 administration). Interestingly, this inhibitory effect of distant tumor was also related to ferroptosis pathway demonstrated by the attenuated inhibition of tumor growth in “DFO + RSL3 + PHT” group when pretreatment with the ferroptosis inhibitor DFO (50 µL, 400 × 10^−6^ m) before 2 h of RSL3 injection (Figure 5b). These results demonstrated that the highly immunogenic and efficient ferroptosis induced in primary tumor can effectively provoke a systemic and specific antitumor effect to prevent the distant tumor growth. Further immune cell analyses were conducted to assess the proportion of CD8^+^ T cells in distant tumor on day 10. As shown in Figure 5c,d, the percentage of CD8^+^ T cells in “RSL3 + PHT” group is 2.7, 1.7, 2.0, and 1.4 times more than that of “Control,” “RSL3,” “PHT,” and “DFO + RSL3 + PHT” groups, respectively, manifesting that massive CD8^+^ T cells can be recruited into the distant untreated tumors after locally evoking highly immunogenic ferroptosis on primary tumor, which mainly contributed to the growth delay of untreated tumor. Consequently, locally evoking highly immunogenic ferroptosis can efficiently trigger a CD8^+^ T cell‐mediated systemic antitumor immunity, displaying a great potential for achieving satisfactory systemic antitumor effect in ferroptosis‐driven cancer therapy.

Finally, the mechanism of realizing satisfactory therapeutic effect through evoking highly immunogenic ferroptosis aided by PHT was proposed in Figure 5e. Herein, TPA‐NDTA NPs were employed to generate PHT, which can not only amplify the ferroptosis efficacy, but also highly improve the immunogenicity during ferroptotic cancer cell death. The enhanced ferroptosis efficacy to 4T1 cancer cells helps reduce the tumor burden and provides a more favorable environment for subsequent immune attack. More importantly, dying cancer cells undergoing highly immunogenic ferroptosis emit massive DAMPs such as ecto‐CRT and ATP. These DAMPs activate the tumor antigen uptake of dendritic cells in tumor site and promote DC maturation and antigen presentation to naïve CD8^+^ T cells in lymph node, which were followed by expanding, differentiating, and migrating into the primary and distant tumors to durably attack the residue cancer cells, tremendously improving the ultimate therapeutic effect in cancer treatment.

## 3 Conclusion

In summary, a novel photothermal molecule TPA‐NDTA was designed by introducing of molecular rotor with strong electron‐donating ability and encapsulated into NPs using DSPE‐PEG_2000_ as the matrix. By virtue of the strong electron‐donating property of molecular rotor and efficient excited‐state intramolecular motion, high‐performance NPs with superb photothermal conversion capability, outstanding stability, and long and broad absorption were developed. After obtaining the NPs, we investigated the highly promoting effect of immunogenicity and efficacy of RSL3‐triggered ferroptosis with the assistance of optimized PHT based on the NPs in 4T1 cancer cells. Next, we conducted in vivo antitumor experiments in a mouse model bearing poor immunogenic 4T1 tumor to evaluate the therapeutic effect through evoking highly immunogenic ferroptosis aided by PHT. Moreover, in the mice with bilateral tumors, locally triggering highly immunogenic ferroptosis on one tumor can prime an efficient CD8^+^ T cell‐mediated systemic antitumor immunity to inhibit the other untreated tumor growth, benefiting a lot to the ultimate outcome of durable and systemic antitumor effect. In this study, we first demonstrated that evoking highly immunogenic ferroptosis aided by a simple but powerful PHT plays a critical role for receiving significantly elevated therapeutic benefits and systemic antitumor capability in ferroptosis‐driven cancer therapy.

## Conflict of Interest

The authors declare no conflict of interest.

## Supporting information

Supporting Information

## Data Availability

The data that support the findings of this study are available from the corresponding author upon reasonable request.
