# Supplementary material for: Evoking Highly Immunogenic Ferroptosis Aided by Intramolecular Motion‐Induced Photo‐Hyperthermia for Cancer Therapy
Source: Adv Sci (Weinh). 2022 Feb 8;9(10):2104885. doi: 10.1002/advs.202104885 (PMC8981454; doi:10.1002/advs.202104885)
Supplement: Supplementary file 1 — Supporting Information [file ADVS-9-2104885-s001.pdf]

WILEY-VCH

ADVANCED SCIENCE
Open Access

Supporting Information

for *Adv. Sci.*, DOI: 10.1002/advs.202104885

Evoking highly immunogenic ferroptosis aided by intramolecular motion-induced photo-hyperthermia for cancer therapy

Chao Chen, Zaiyu Wang, Shaorui Jia, Yuan Zhang, Shenglu Ji, Zheng Zhao, Ryan T. K. Kwok, Jacky W. Y. Lam, Dan Ding, Yang Shi* and Ben Zhong Tang**

Supporting Information

Evoking highly immunogenic ferroptosis aided by intramolecular motion-induced photo-hyperthermia for cancer therapy

Chao Chen, Zaiyu Wang, Shaorui Jia, Yuan Zhang, Shenglu Ji, Zheng Zhao, Ryan T. K. Kwok, Jacky W. Y. Lam, Dan Ding, Yang Shi* and Ben Zhong Tang**

1. Experimental section

Materials: All chemical reagents were obtained from Sigma-Aldrich unless otherwise stated. MTT (3-(4,5)-dimethylthiahiazo (-z-y1)-2,5-di-phenytetrazoliumromide), DCFH-DA (2',7'-dichlorodihydrofluorescein diacetate) and DAPI (4',6-diamidino-2-phenylindole) were also purchased from Sigma-Aldrich. Lipid peroxidation sensor (C11 BODIPY 581/591) and fluorescent secondary antibodies for immunofluorescence staining was bought from Invitrogen. Lipid peroxidation (MDA) assay kit and primary antibodies were provided by Abcam. ATP assay kit was offered by Beyotime. RPMI 1640 cell culture medium, penicillin-streptomycin (PS) and fetal bovine serum (FBS) were purchased from BioInd. Directly-labeled primary antibodies for flow cytometric analysis were provided by Biolegend. RSL3 ((1S,3R)-RSL3) and deferoxamine (DFO) were purchased from Selleck. 4T1 mouse breast cancer cells were purchased from ATCC.

Characterization: ^{1}H and ^{13}C NMR were determined using a Bruker-DPX 400 spectrometer. HRMS (High-resolution mass spectra) were conducted by a Varian 7.0 T FTMS Mass Spectrometer System under the condition of electrospray ionization (ESI). UV-vis spectra were measured using a Shimadzu UV-1700 spectrometer. Photoluminescence (PL) spectra were measured through Edinburgh FS5 fluorescence spectrophotometer and Milton Roy Spectronic 3000 array spectrophotometer. The morphology and diameter of TPA-NDTA NPs were performed by transmission electron microscopy (TEM, JEM-2010FJEOL, Japan) and dynamic light scattering (90 Plus,

Brookhaven Instruments Co. USA). Photoacoustic imaging in vitro and in vivo were conducted using MOST imaging system (inVision 256-TF; iThera Medical, Germany).

Synthesis of compound NDTA: Under a N_2 atmosphere, a mixture of NDI-4Br (1.14 g, 1 mmol) and sodium 1,1-cyanoethylene-2,2-dithiolate hydrate (700 mg, 3 mmol) in THF (30 mL) was stirred at room temperature for 3 hours. The solvent was removed under reduced pressure. Chromatography of the residue on a silica gel column with chloroform/hexane (1/2) as eluent afforded 940 mg NDTA as a blue solid (yield: 90%) ^{1}H NMR (400 MHz, Chloroform-*d*) δ 5.66 (s, 2H), 4.15 (d, J = 7.4 Hz, 4H), 1.97 (q, J = 6.4 Hz, 2H), 1.41 – 1.20 (m, 64H), 0.86 (q, J = 6.0 Hz, 12H). ^{13}C NMR (101 MHz, Chloroform-*d*) δ 164.62, 162.22, 162.07, 115.93, 115.64, 84.89, 36.48, 31.94, 31.90, 31.51, 30.03, 29.67, 29.65, 29.59, 29.54, 29.37, 29.32, 22.69, 14.12.

Synthesis of compound NDTA-2Br: To a solution of NDTA (940 mg, 0.9 mmol) in $CHCl_3$ (30 mL), Br_2 (320 mg, 2 mmol) in 1 mL $CHCl_3$ was added dropwise at room temperature, and the mixture was stirred for 2 hours. The solvent was then removed under reduced pressure. The residue was purified by column chromatography with chloroform/hexane (1/2) as eluent and 1.03 g NDTA-2Br was obtained in a yield of 92%. ^{1}H NMR (400 MHz, Chloroform-*d*) δ 4.18 (dt, J = 7.6, 4.0 Hz, 4H), 2.03 (dq, J = 14.5, 7.3, 6.8 Hz, 2H), 1.40 – 1.21 (m, 64H), 0.86 (td, J = 6.7, 3.6 Hz, 12H). ^{13}C NMR (101 MHz, Chloroform-*d*) δ 162.03, 161.68, 116.55, 114.26, 71.27, 46.21, 36.46, 31.94, 31.91, 31.51, 30.09, 29.70, 29.67, 29.63, 29.58, 29.38, 29.35, 26.34, 22.70, 14.13.

Synthesis of compound TPA-NDTA: To a solution of NDTA-2Br (121 mg, 0.1mmol), MeOTPA-B(OH)$_2$ (175 mg, 0.5 mmol) and K_2CO_3 (111 mg, 0.8 mmol) in THF (10 mL) and H_2O (3 mL), Pd(PPh$_3$)$_4$ (11.6 mg, 0.01 mmol) was added under N_2 protection. The mixture was stirred overnight at 100 °C under N_2 protection, after which the mixture was extracted with H_2O/CH_2Cl_2 for three times and the organic layer was collected and organic solvent was removed under reduced pressure. The crude product was purified

by column chromatography to afford pure 2MeOTPA-NDTA (124 mg) in a yield of 75%. ^{1}H NMR (400 MHz, Chloroform-*d*) δ 7.29 (t, J = 7.8 Hz, 4H), 7.14 (d, J = 8.3 Hz, 8H), 6.99 – 6.78 (m, 12H), 4.14 (d, J = 7.5 Hz, 4H), 3.83 (d, J = 1.9 Hz, 12H), 2.05 (d, J = 5.4 Hz, 2H), 1.38 – 1.16 (m, 64H), 0.87 – 0.80 (m, 12H). ^{13}C NMR (101 MHz, Chloroform-*d*) δ 162.10, 162.03, 161.87, 156.84, 156.82, 151.94, 149.22, 147.20, 139.47, 139.43, 127.84, 127.78, 127.72, 123.09, 118.35, 118.30, 117.00, 115.23, 114.99, 101.32, 55.52, 46.21, 36.23, 31.95, 31.91, 31.87, 31.57, 31.47, 31.36, 30.26, 30.23, 30.20, 29.75, 29.72, 29.68, 29.66, 29.62, 29.40, 26.56, 26.47, 22.70, 14.13. HRMS (MALDI-TOF): m/z: $[M+H]^+$ calcd for $C_{100}H_{118}N_6O_8S_4$, 1659.7972; found, 1659.7990.

Theoretical calculation: The molecule was optimized with the density functional theory method by using B3LYP density functional and Def2-SVP basis set. London-dispersion effects were also taken into consideration using Grimme’s DFT-D3 correction, to further describe long-range intramolecular interactions.[1] Analytical frequency calculations were also performed at the same level of theory to confirm that the optimized structures were at a minimum point. Above quantum chemical calculations were carried out by using Gaussian 16 program.[2] The electron cloud distribution maps were also displayed using IQmol molecular viewer package.

Preparation of TPA-NDTA NPs: Briefly, 1 mg of TPA-NDTA and 3 mg of DSPE-PEG_{2000} were dissolved in 1mL of THF, which was followed by dropping into 9 mL of water under ultrasonication using a microtip probe sonicator (XL2000, Misonix Incorporated, NY). After addition of the mixture solution, 5 min of ultrasonication was further performed. Then, removing the THF solvent by dialysis using ddH_2O and concentrating to 1 mg/mL (based on TPA-NDTA) through ultrafiltration (5000 × g for 15 min).

Cell culture: 4T1 mouse breast cancer cells were incubated with complete RPMI 1640 culture medium including 10% FBS (fetal bovine serum) and 1% PS (penicillin streptomycin) in artificial environment of 5% CO_2, 37 ^{o}C and certain humidity. The

cells were passaged every three days when the cell density reaches to 90%.

Animal and tumor-bearing mouse model: All animal experiments in this work were performed under the guidelines set by Tianjin Committee of Use and Care of Laboratory Animals, and the overall project protocols were approved by the Animal Ethics Committee of Nankai University. The healthy female BALB/c mice were purchased from the Laboratory Animal Center of the Academy of Military Medical Sciences (Beijing, China). 1×10^5 live 4T1 cancer cells were injected into the right axilla to establish tumor-bearing mice for evaluate the antitumor effect by boosting the immunogenicity and efficacy of RSL3 in assistant of photo-hyperthermia based on TPA-NDTA NPs. After 14 days, when the tumor size reached to ~250 mm^3, the corresponding treatments were performed according to the design. The tumor volume was calculated by the following equation: Volume = $Width^2 \times Length/2$. Mice were euthanized when the tumor volume over 1500 mm^3. For establishing the mouse model of bilateral tumor, the mice were subcutaneously injected with 1×10^5 live 4T1 cancer cells into the right axilla. After 7 days, all mice were received the second tumor inoculation in the left axilla using the same 4T1 cancer cells (1×10^5). Then, the primary tumors of the mice in 5 groups were received corresponding treatments according to design and the secondary tumor growth was monitored by caliper.

Lipid peroxidation detection in vitro and in vivo: For in vitro detection, 4T1 cancer cells were seeded in the special confocal champers and treated according the designed protocols. In brief, after adherence, the cells were divided into five groups, named "Control", "RSL3", "PHT", "RSL3 + PHT" and "DFO + RSL3 + PHT", respectively. After corresponding treatments, all the cells were incubated with 10 μM of C11 BODIPY 581/591 for 30 min, then taken imaging using CLSM. 488 nm of the excitation was used, and the collection range is from 505 to 550 nm. The signals collected in this condition indicate the levels of oxidized lipid. For in vivo determination, another method (Lipid peroxidation (MDA) assay kit) was used to

evaluate the levels of lipid peroxidation. Malondialdehyde (MDA) is a natural product of lipid oxidation that will accumulate after suffering the oxidization in vivo. Thus, the amount of MDA reveals the levels of lipid peroxidation in vivo. After relevant treatments in tumor-bearing mice of 5 groups, the tumors were collected and prepared according to the instruction. This kit will allow the quantitation of MDA adduct in protein samples determined by comparing its absorbance at 450 nm with that of a known MDA-BSA standard curve.

ecto-CRT staining in vitro and in vivo: 4T1 cancer cells were seeded in the special confocal champers and divided into 5 groups, named “Control”, “RSL3”, “PHT”, “RSL3 + PHT” and “DFO + RSL3 + PHT”, respectively. After 12 h of corresponding treatments of rational design mentioned in main text, the cells were washed with 1×PBS and fixed using 4% paraformaldehyde for 20 min at 4 ^{o}C, which were incubated with 1×PBS solution containing anti-calreticulin primary antibodies (1:200 dilution in 1×PBS) overnight at 4 ^{o}C in a humidor after washing three times by 1×PBS. After returning to room temperature, the cells were washed three times by 1×PBS carefully and incubated with the Alexa Fluor 488-conjugated secondary antibodies (1:200 dilution in 1×PBS) for 2 h at room temperature. Subsequently, DAPI containing PBS solutions were replaced into the champers and incubated for another 15 min. Finally, CLSM was applied to investigate the expression levels of ecto-CRT in these 5 groups. The laser excitation is 488 nm and the signals were collected within the range from 500 nm to 600 nm. For in vivo analysis of ecto-CRT, the tumors in 5 groups after corresponding treatments were fixed using 4% paraformaldehyde for 24 h at 4 ^{o}C. Then, 20%, 30% and 40% of sucrose solutions were prepared, and the fixed tumors were successively incubated in these sucrose solutions for 3 days according to the order of increasing concentration (one day for each solution). After that, the tumor tissues were embedded into OCT (opti-mum cutting temperature compound) mixture and fabricated into frozen sections with the thickness of 8 μm by cryostats (Leica CM1950) for further immunofluorescence staining. Subsequently, the tumor frozen sections of 5 groups

were first washed three times and incubated with goat serum blocking solution for 20 min at room temperature, which were then incubated with primary antibody solutions (anti-calreticulin antibodies) overnight at 4 ^{o}C in a humidor. After returning to room temperature, the samples were incubated with the secondary antibody solutions (Alexa Fluor 633-conjugated secondary antibody) for 2 h at room temperature in a humidor after washing three times by 1×PBS, which then treated with DAPI fluoromount. After drying, the samples were imaged by CLSM with the excitation of 633 nm and collection from 650 nm to 750 nm.

ROS study in 4T1 cancer cells: 4T1 cancer cells were seeded in the special confocal champers and divided into 5 groups, named “Control”, “RSL3”, “PHT”, “RSL3 + PHT” and “DFO + RSL3 +PHT”, respectively. After adherence, the cells were treated separately according to the designed procedures. After 12 h of the corresponding treatments, the cells were incubated with 25 μM of DCFH-DA for 45 min, then washed three times by 1×PBS and replaced with fresh cell culture medium. Once addition of DCFH-DA into the medium, the incubation and operation need avoid light irradiation. Finally, the cells were taken images using CLSM under 488 nm excitation, and the signals were collected from 500 nm to 550 nm.

PA imaging in vitro and in vivo: 50 μg/mL of TPA-NDAT NPs solution was used to measure the PA spectrum from 680 nm to 900 nm using a commercial small-animal optacoustic tomography system (MOST, iTheraMedical, Germany). A series of concentrations of TPA-NDTA NPs solutions were prepared to determine the PA signals under the excitation of 810 nm. The PA signals of ICG and TPA-NDTA NPs solutions with the same molar concentration were also compared under the 810 nm excitation using this PA imaging system. The PA images were reconstructed from the software of this machine. For in vivo PA imaging, the tumor-bearing mice were anesthetized by 2% isoflurane in oxygen and intravenously injected with 50 μg/mL of TPA-NDTA NPs (100 μL). The PA images of the mice were collected at the designed time points after

injection under the excitation of 810 nm.

Ex vivo analysis of immune cells: In order to investigate the immune mechanism of the anti-tumor effect by boosting the immunogenicity and efficacy of ferroptosis with the help of photo-hyperthermia, relevant immune cells were analyzed using flow cytometric. In brief, the mice of 5 groups including “Control”, “RSL3”, “PHT”, “RSL3 + PHT” and “DFO + RSL3 + PHT” were sacrificed after 48 h of corresponding treatments, the tumor-draining lymph nodes were collected and prepared to single-cell suspensions by mechanical grinding, then incubated with the mixed antibody solutions (including anit-CD11c-PE antibodies, anti-CD80-APC antibodies and anti-CD86-FITC antibodies) for 15 min in dark and subsequently determined using flow cytometric after wash twice. For evaluation of $CD8^+$ T cells of the tumor tissues, the mice were sacrificed and the tumors were harvested and prepared into single-cell suspensions by mechanical dissociation after 5 days of different treatments. Subsequently, the tumor samples of 5 groups were treated with red blood cell lysis buffer for 10 min at room temperature to remove red blood cells, which influence the fluorescence staining. After that, the samples were all co-stained with anti-CD3-FITC antibodies and anti-CD8-APC antibodies for 15 min in dark, and analyzed using flow cytometric after wash twice. In terms of the bilateral tumor-bearing mice, the primary tumors of the mice in 5 groups were received corresponding treatments after establishing the secondary tumors. On day 10, all the mice of 5 groups were sacrificed and the secondary tumors were collected and prepared into single-cell suspensions through mechanical dissociation, which were followed by purified through removing the red blood cells and co-stained with anti-CD3-FITC and anti-CD8-APC for further flow cytometric analysis.

Statistical Analysis: Mean ± standard deviation (SD) was used for quantitative data analysis. Statistical comparisons in the 5 groups were analyzed by one-way ANOVA with a Tukey post-hoc test. P value < 0.05 means statistically significant difference.

2. Supporting Figures

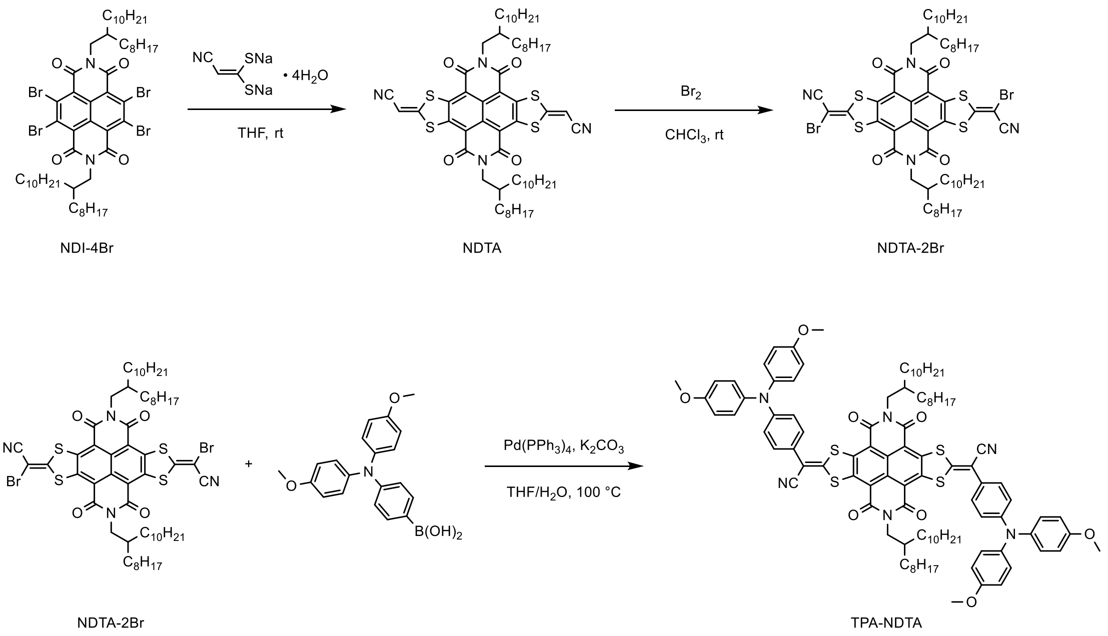

Figure S1. Synthetic route to TPA-NDTA.

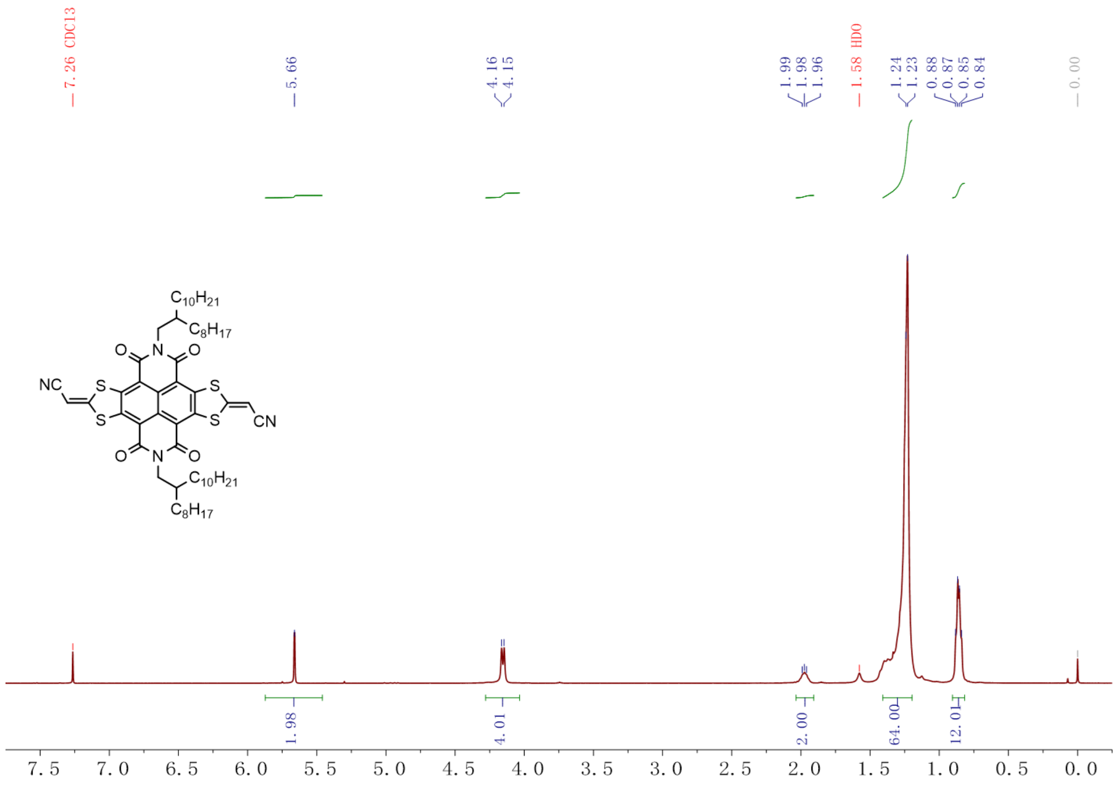

Figure S2. ^{1}H NMR spectrum of NDTA.

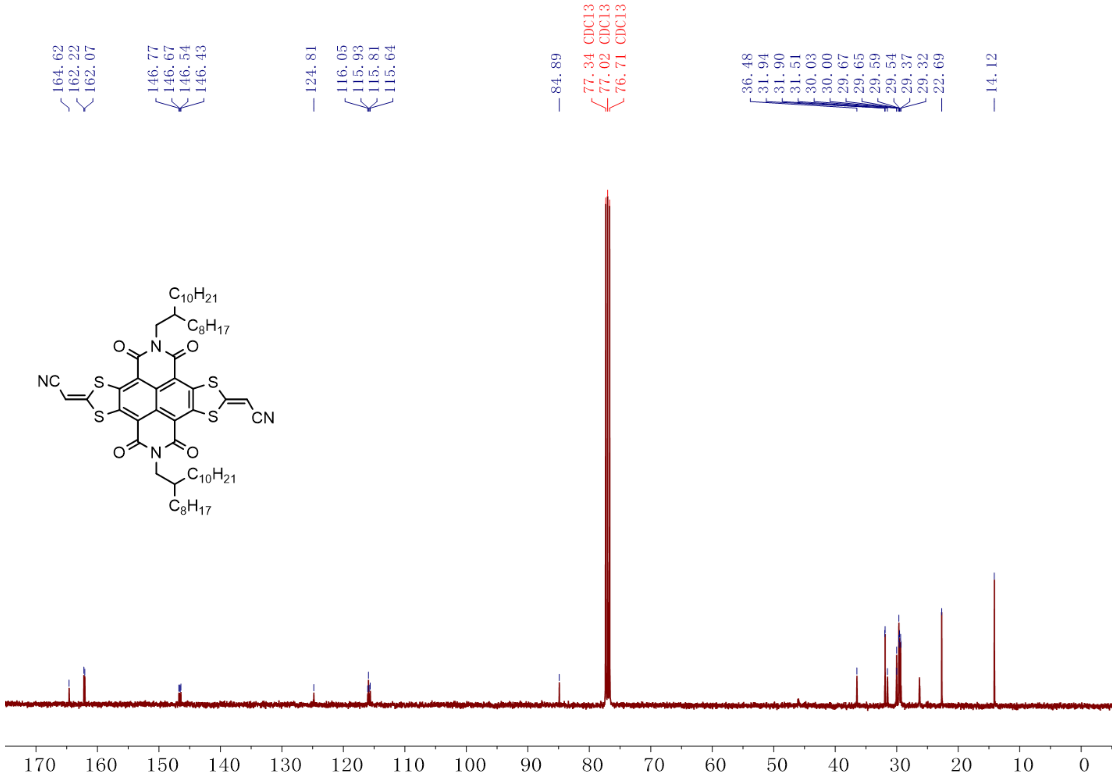

Figure S3. ^{13}C NMR spectrum of NDTA.

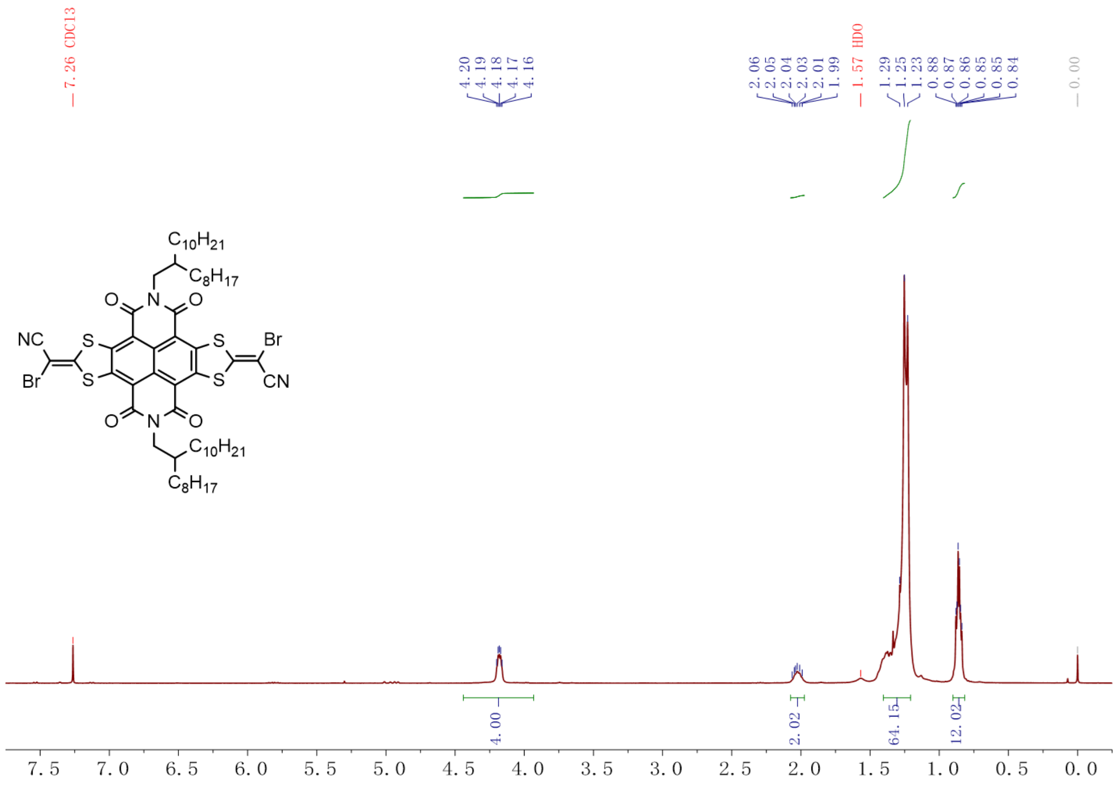

Figure S4. ^{1}H NMR spectrum of NDTA-2Br.

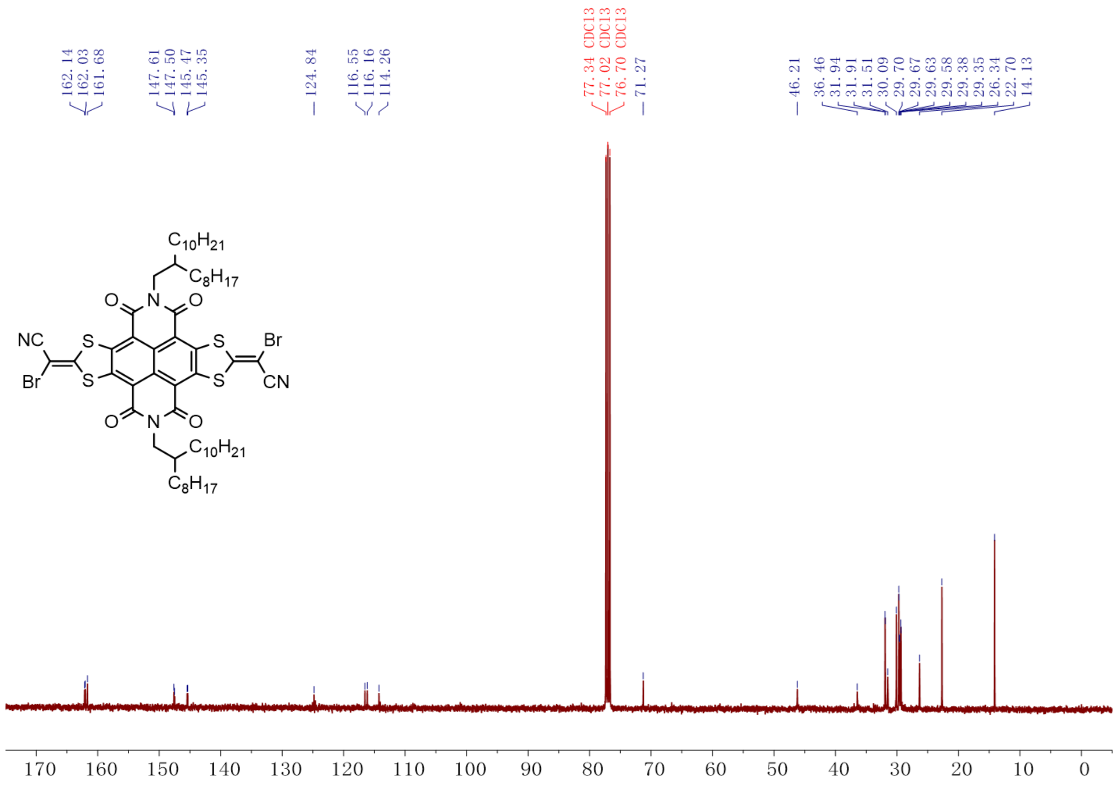

Figure S5. ^{13}C NMR spectrum of NDTA-2Br.

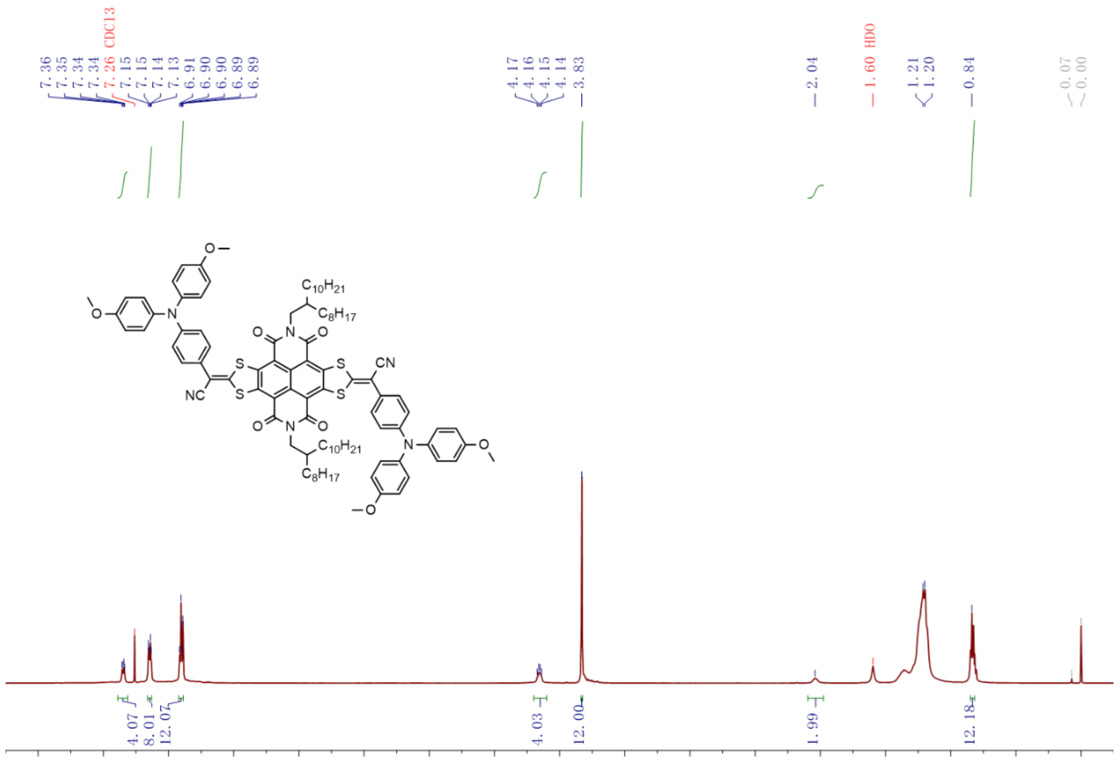

Figure S6. ^{1}H NMR spectrum of TPA-NDTA.

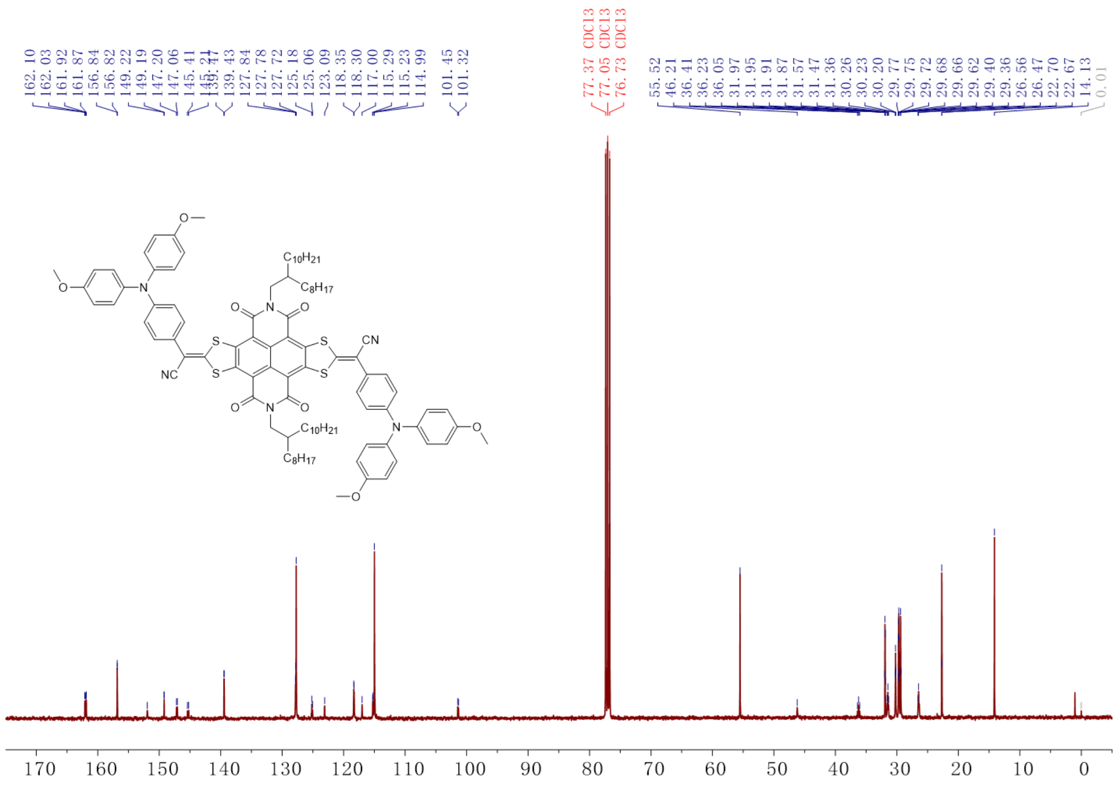

Figure S7. ^{13}C NMR spectrum of TPA-NDTA.

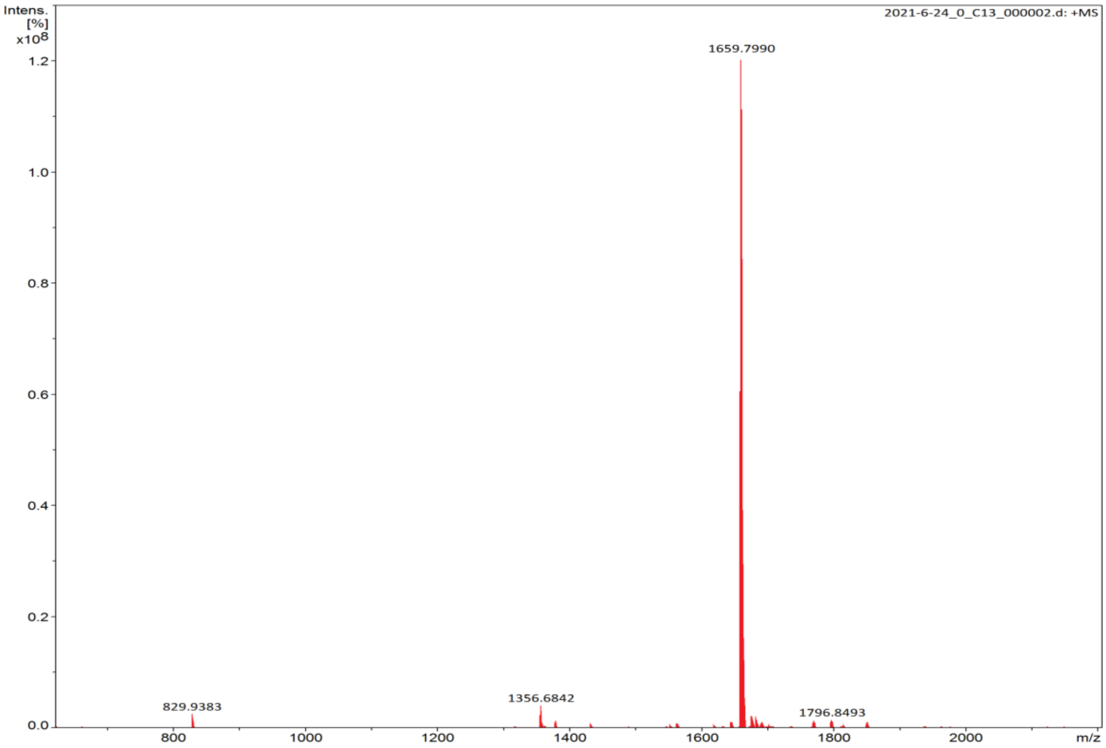

Figure S8. HRMS (ESI) spectrum of TPA-NDTA.

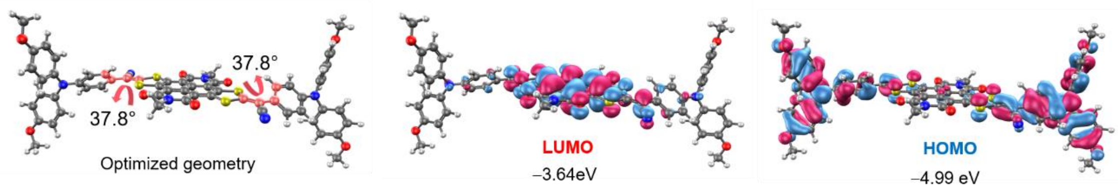

Figure S9. Optimized ground state geometry and electron cloud distributions of the highest occupied molecular orbital (HOMO) and the lowest unoccupied molecular orbital (LUMO), calculated by DFT methods at M06-2X/Def2-SVP level, Gaussian 16 program. Alkyl chains are simplified as the methyl group for clarity which doesn't affect the calculated result.

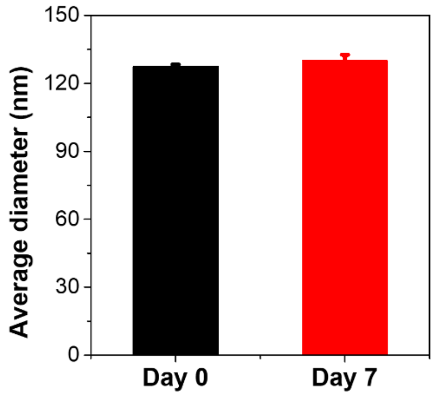

Figure S10. Average diameter of the NPs on day 0 and day 7 in PBS detected using DLS. Concentration: 50 µg/mL based on TPA-NDTA molecules. *n* =3 for each group.

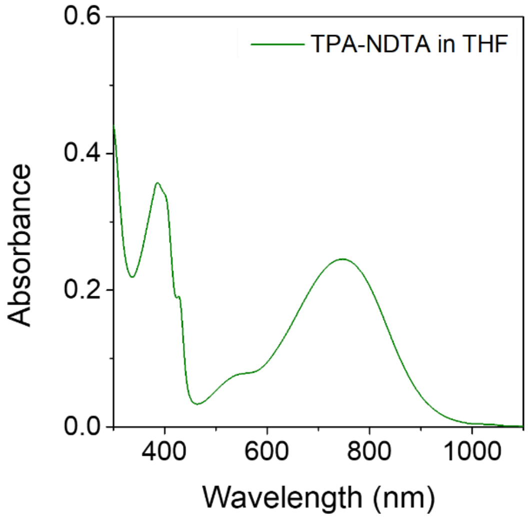

Figure S11. UV-vis absorbance spectrum of TPA-NDTA (10 µM) in THF.

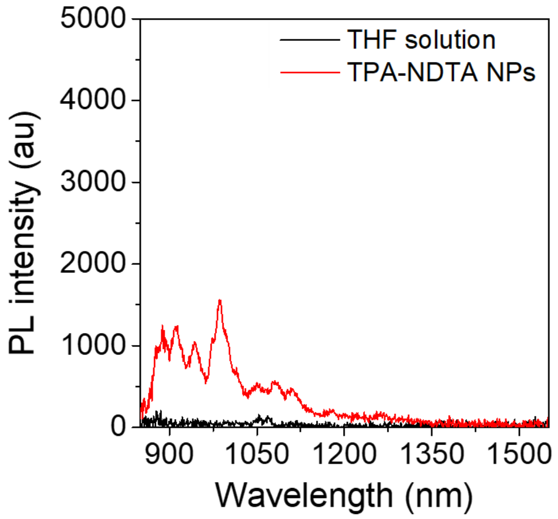

Figure S12. PL spectra of TPA-NDTA in THF solution and within nanoparticle (DSPE-PEG_{2000} as the matrix, in water) based on the same mass concentration of TPA-NDTA.

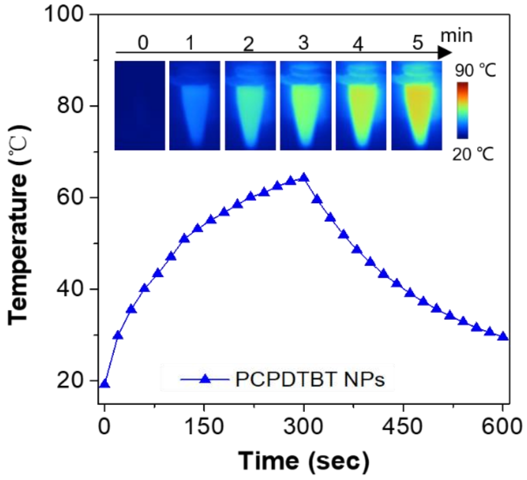

Figure S13. The photothermal conversion performance of PCPDTBT NPs under 808 nm laser irradiation for 5 min at 0.8 W/cm^2. The cooling process is spontaneous cooling. Insert photos are the IR thermal images of different time points including 0, 1, 2, 3, 4, 5 min.

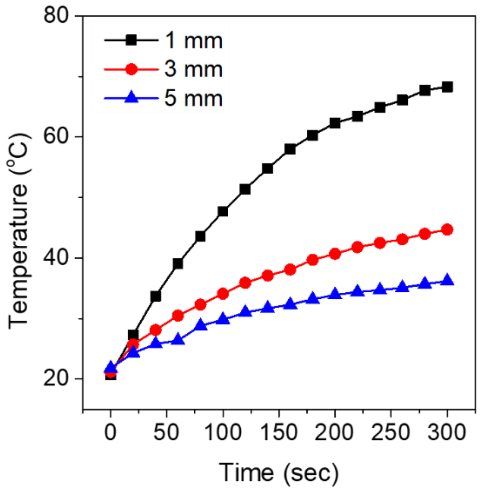

Figure S14. The plots of temperature increase against irradiation time under 808 nm laser irradiation (0.8 W/cm^2) when covering by chicken tissues with different thickness (1, 3 and 5 mm).

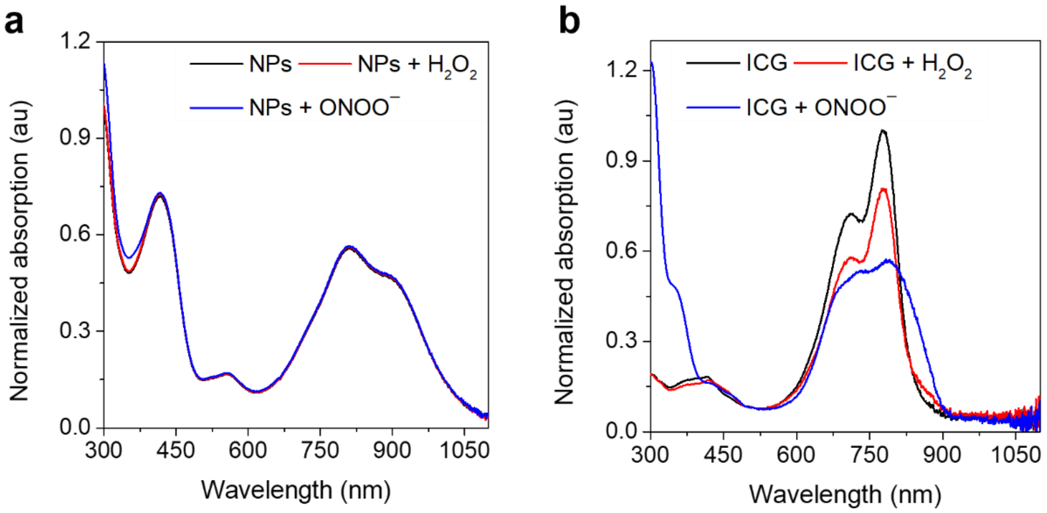

Figure S15. Normalized absorption spectra of a) TPA-NDTA NPs solutions (12.5 μg/mL based on TPA-NDTA) and ICG solutions (12.5 ug/mL) with or without 400 μM of $ONOO^-$ or H_2O_2, respectively. The absorbance of the NPs/ICG solutions added with $ONOO^-$ and H_2O_2 is measured after 5 min of reaction.

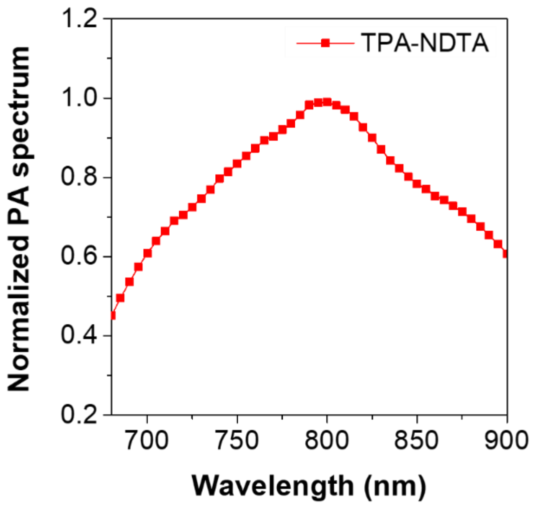

Figure S16. Normalized PA spectrum of TPA-NDTA NPs solution (50 μg/mL) under 810 nm excitation.

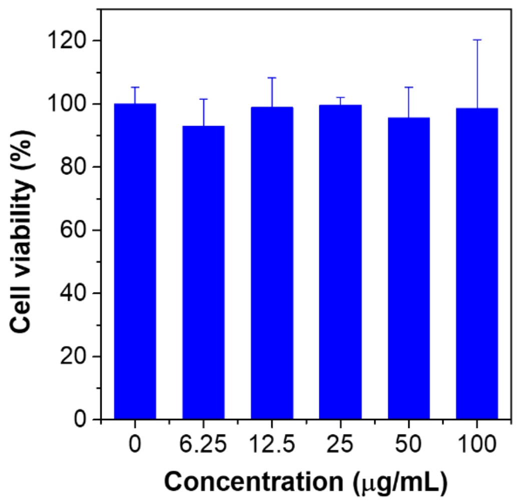

Figure S17. Cell viability of 4T1 cancer cells incubated with different concentrations of TPA-NDTA NPs for 24 h without laser irradiation. *n* = 3 for each group.

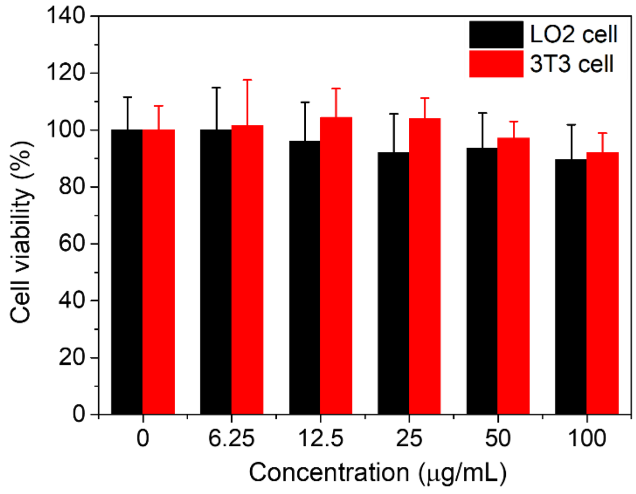

Figure S18. The cell viabilities of mouse fibroblast line 3T3 and human hepatocyte cell line LO2 were measured using MTT assay after incubation with a series of NP concentrations for 72 h. n = 3 for each group.

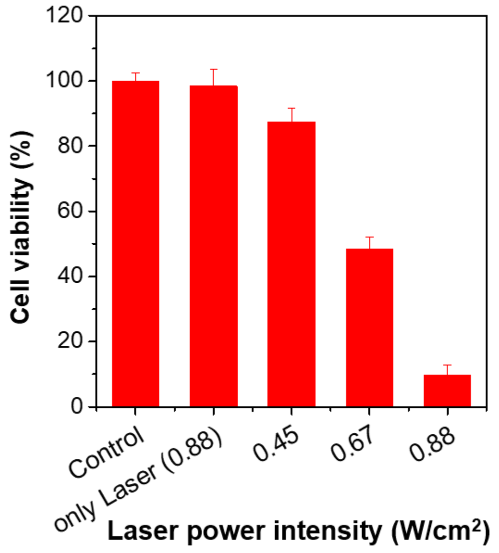

Figure S19. Cell viability of 4T1 cancer cells exposed to laser irradiation with different power intensities for 5 min after incubation with the same concentration of TPA-NDTA NPs (25 μg/mL). n = 3 for each group.

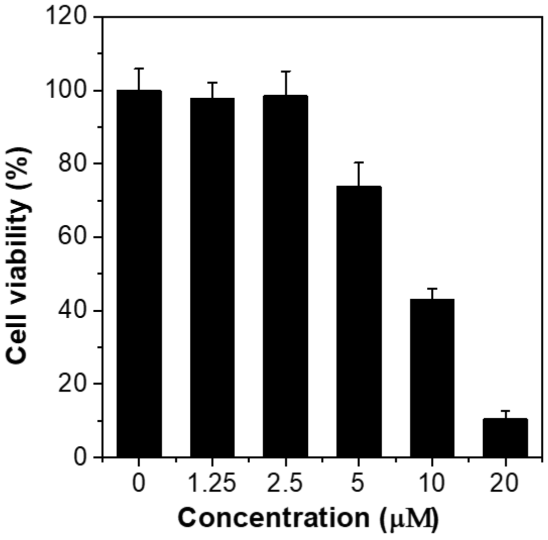

Figure S20. Cell viability of 4T1 cancer cells incubated with different concentrations of RSL3 for 24 h. n = 3 for each group.

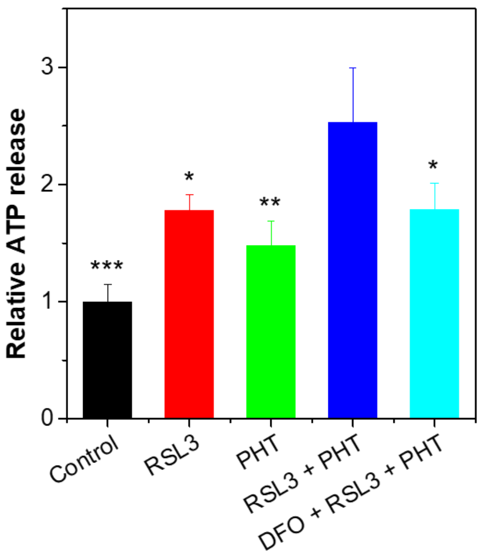

Figure S21. Relative ATP release of 4T1 cancer cells after different treatments (n = 3). Statistical significance was determined by one-way ANOVA with a Tukey post-hoc test. $^{*}P < 0.05$, $^{**}P < 0.01$, $^{***}P < 0.001$, in comparison with "RSL3 + PHT" group.

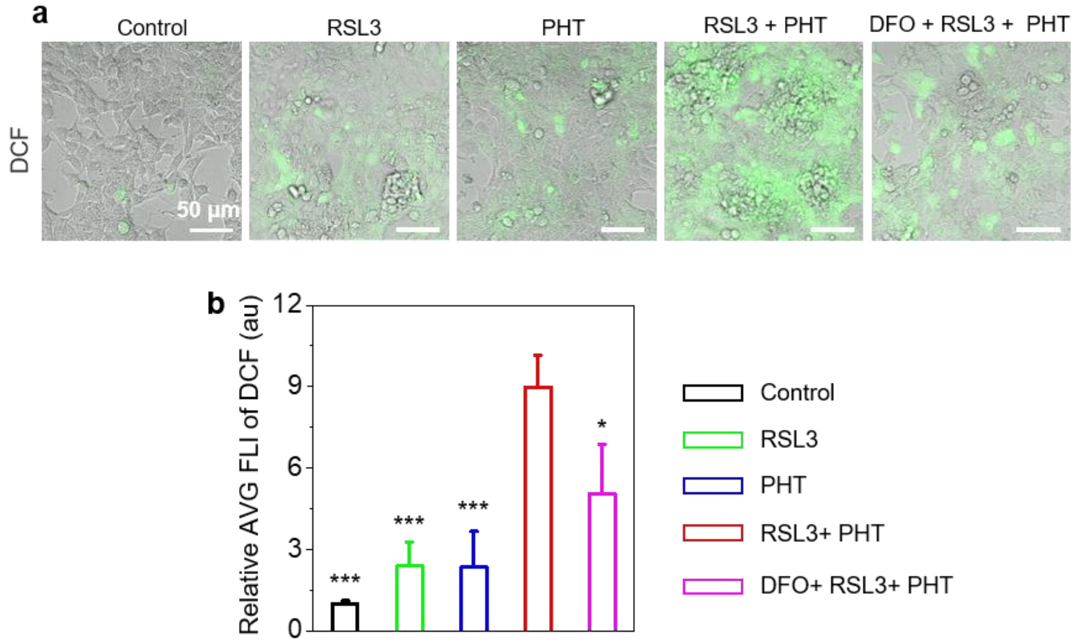

Figure S22. Cellular ROS detection using DCF-DA as the indicator. (a) Representative CLSM images of cellular ROS in five groups after corresponding treatments at 12 h. The intensity of green signal indicates the levels of cellular ROS. (b) Quantitative analysis of CLSM images of cellular ROS in five groups (n = 3). Statistical significance was determined by one-way ANOVA with a Tukey post-hoc test. $^{*}P < 0.05$, $^{***}P < 0.001$, in comparison with "RSL3 + PHT" group.

**Blood routine examination**		**Control group**		**Experimental group**	
items	units	Average value	Standard Deviation	Average value	Standard Deviation
white blood cell	10^9/L	5.636666667	1.148448228	6.162333333	1.296613409
red blood cell	10^{12}/L	10.84666667	0.458839115	9.892166667	0.280880906
hemoglobin	g/L	165	4.582575695	162.45	14.88816644
Hematocrit	%	59.93666667	2.100960098	52.36666667	1.650252506
mean corpuscular volume	fL	56.3	3.46554469	53.83833333	1.708334959
mean corpuscular hemoglobin	pg	15.46666667	0.611010093	15.20166667	0.438757716
mean corpuscular hemoglobin concentration	g/L	280	10.58300524	287.4833333	10.23478546
Platelet	10^9/L	203	89.4483091	208.8	6.238589584
red blood cell distribution width-SD	fL	25.7	1.212435565	26.975	0.815092019
red blood cell distribution width-CV	%	19.68666667	1.797368447	20.29	0.607700584
platelet distribution width	fL	8.433333333	0.404145188	9.455	0.268467875
**Liver function tests**		**Control group**		**Experimental group**	
items	units	Average value	Standard Deviation	Average value	Standard Deviation
blood urea	mmol/L	5.710333333	0.168790798	6.409666667	0.843095685
creatinine	umol/L	17.81666667	0.742181469	15.53333333	4.500370355
albumin	g/L	39.54666667	1.473273	39.48	1.145949388
alanine aminotransferase	U/L	45.525	10.97448746	39.77333333	7.755264878
aspartate aminotransferase	U/L	117.1216667	16.96798186	117.42	9.59401897
total bilirubin	umol/L	1.207	0.260282539	1.493	0.58721972
alkaline phosphatase	U/L	154.3666667	17.97229349	143.6333333	5.822656896

Figure S23. Results of toxicity detection of TPA-NDTA NPs in vivo after tail vein

injection including blood routine examination and liver function tests. The results indicate that there is no obvious difference after intravenous injection of TPA-NDTA comparing with the healthy mice in control group without any treatment. Control group: healthy mice without any treatment; Experimental group: TPA-NDTA injection (i.v.). n = 3 for each group.

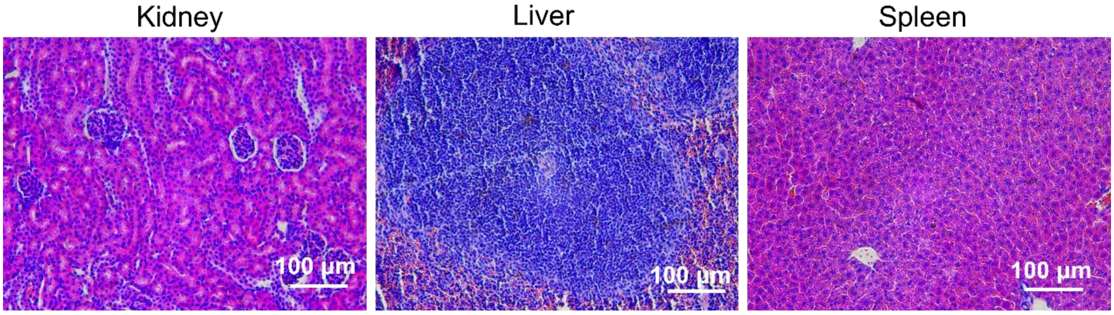

Figure S24. Representative H&E staining of kidney, liver and spleen of the healthy mice post 7 days of intravenous administration by 100 μL of TPA-NDTA NPs solution (200 μg/mL in 1 × PBS). n = 3 for each group.

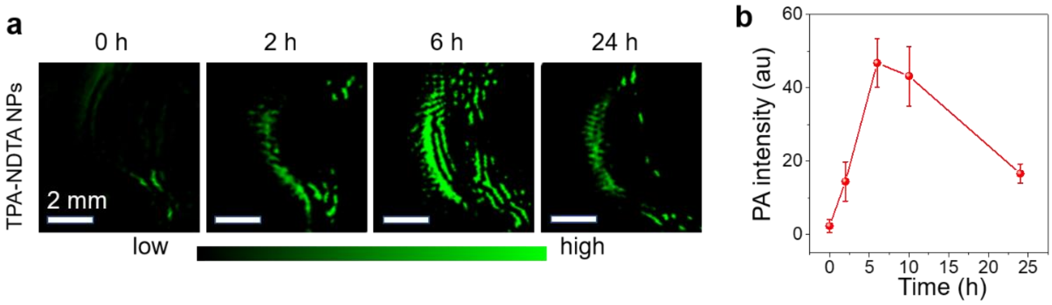

Figure S25. (a) Representative PA images of 4T1 tumor of the mice at different time points after intravenous administration of TPA-NDTA NPs (100 μL, 50 μg/mL). (b) Quantitative analysis of these PA images. n = 3 for this imaging experiment.

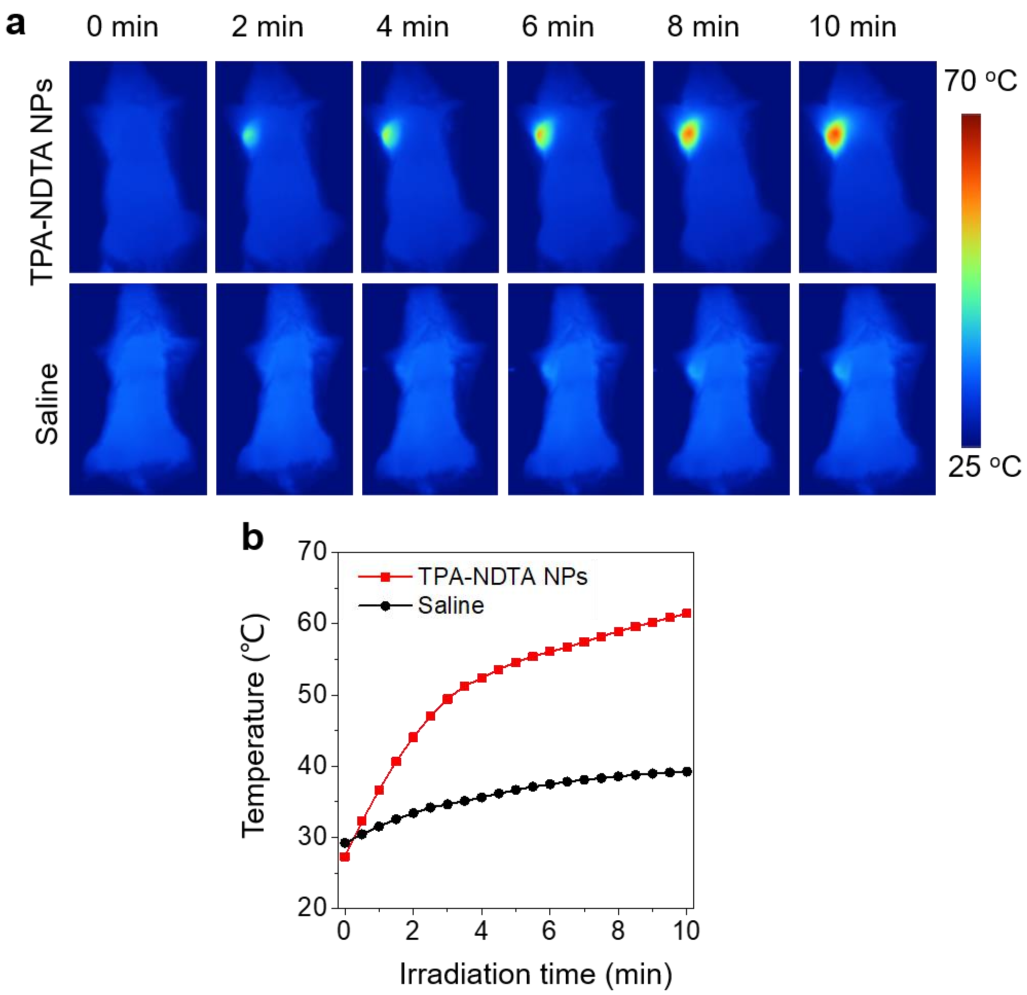

Figure S26. (a) IR thermal images of tumor-bearing mice exposed to local laser irradiation (808 nm, 0.6 W/cm^2) for different times after 6 h of intravenous injection of TPA-NDTA NPs (100 μL, 50 μg/mL) or saline, respectively. (b) Plot of the mean temperature of the 4T1 tumors vs. local laser irradiation (808 nm, 0.6 W/cm^2) times post 6 h of NPs/saline injection in two groups.

Reference:

[1] Goerigk, L.; Hansen, A.; Bauer, C.; Ehrlich, S.; Najibi, A.; Grimme, S. A Look at the Density Functional Theory Zoo with the Advanced GMTKN55 Database for General Main Group Thermochemistry, Kinetics and Noncovalent Interactions. *Phys. Chem. Chem. Phys.*, 2017, 19, 32184-32215.

[2] M. J. Frisch, G. W. Trucks, H. B. Schlegel, G. E. Scuseria, M. A. Robb, J. R.

Cheeseman, G. Scalmani, V. Barone, G. A. Petersson, H. Nakatsuji, X. Li, M. Caricato, A. V. Marenich, J. Bloino, B. G. Janesko, R. Gomperts, B. Mennucci, H. P. Hratchian, J. V. Ortiz, A. F. Izmaylov, J. L. Sonnenberg, D. Williams-Young, F. Ding, F. Lipparini, F. Egidi, J. Goings, B. Peng, A. Petrone, T. Henderson, D. Ranasinghe, V. G. Zakrzewski, J. Gao, N. Rega, G. Zheng, W. Liang, M. Hada, M. Ehara, K. Toyota, R. Fukuda, J. Hasegawa, M. Ishida, T. Nakajima, Y. Honda, O. Kitao, H. Nakai, T. Vreven, K. Throssell, J. A. Montgomery, Jr., J. E. Peralta, F. Ogliaro, M. J. Bearpark, J. J. Heyd, E. N. Brothers, K. N. Kudin, V. N. Staroverov, T. A. Keith, R. Kobayashi, J. Normand, K. Raghavachari, A. P. Rendell, J. C. Burant, S. S. Iyengar, J. Tomasi, M. Cossi, J. M. Millam, M. Klene, C. Adamo, R. Cammi, J. W. Ochterski, R. L. Martin, K. Morokuma, O. Farkas, J. B. Foresman, and D. J. Fox, *Gaussian 16*, Gaussian, Inc., Wallingford CT, 2016.
